# Trajectories of Self-Awareness Across the Alzheimer’s Disease Spectrum: A Systematic Review of Its Potential Contribution to Early Diagnosis

**DOI:** 10.3390/diagnostics16172791

**Published:** 2026-08-30

**Authors:** Anastasia Tsouvala, Despina Moraitou, Panagiota Metallidou, Glykeria Tsentidou, Ioanna-Giannoula Katsouri, Georgia Papantoniou, Maria Sofologi, Magdalini Tsolaki

**Affiliations:** 1 Laboratory of Psychology, Department of Cognition, Brain and Behavior, School of Psychology, Aristotle University of Thessaloniki (AUTh), 54124 Thessaloniki, Greece; demorait@psy.auth.gr (D.M.); pmetall@psy.auth.gr (P.M.); gtsentid@psy.auth.gr (G.T.); 2 Laboratory of Neurodegenerative Diseases, Center for Interdisciplinary Research and Innovation, Aristotle University of Thessaloniki (CIRI—AUTh), 54124 Thessaloniki, Greece; tsolakim@auth.gr; 3 Greek Association of Alzheimer Disease & Related Disorders, 54643 Thessaloniki, Greece; 4 Occupational Therapy Faculty, University of West Attica, 12243 Athens, Greece; ykatsouri@uniwa.gr; 5 Laboratory of Psychology, Department of Early Childhood Education, School of Education, University of Ioannina, 45110 Ioannina, Greece; gpapanto@uoi.gr (G.P.); m.sofologi@uoi.gr (M.S.); 6 Institute of Humanities and Social Sciences, University Research Centre of Ioannina (URCI), 45110 Ioannina, Greece; 7 Department of Psychology, School of Health Sciences, Neapolis University Pafos, Pafos 8042, Cyprus

**Keywords:** self-awareness, metacognition, subjective cognitive decline, mild cognitive impairment, Alzheimer’s disease continuum, cognitive performance, neuroimaging markers

## Abstract

**Background/Objectives**: Self-awareness constitutes a key metacognitive construct supporting self-regulation and adaptive functioning in aging. This systematic review examined how self-awareness fluctuates across the Alzheimer’s disease (AD) continuum and explored its associations with cognitive performance and neuroimaging markers. **Methods:** A systematic search was conducted in databases including PubMed, Scopus, Science Direct and Web of Science covering the period from 2016 to 2026, and the review was registered on the Open Science Framework (OSF). The selection process followed PRISMA guidelines, and a total of 334 studies were screened for eligibility while 42 met the inclusion criteria. Studies were eligible if they examined self-awareness in relation to cognitive and/or neuroimaging parameters, with individuals in the preclinical and clinical spectrum of AD as the reference population. **Results:** The included studies highlighted self-awareness as a dynamic construct closely linked to cognitive performance and neural integrity, with measurable deviations emerging along the continuum from subjective cognitive decline to dementia. Accordingly, the findings suggest that alterations in self-awareness may reflect the stage-dependent cognitive and neurobiological changes that characterize the progression of AD. **Conclusions:** Converging evidence suggests that assessing fluctuations of self-awareness, ranging from heightened awareness to reduced awareness, may contribute to the early identification of individuals at risk of progression across the AD continuum. However, the substantial methodological heterogeneity across studies precludes definitive conclusions regarding its clinical utility. Future longitudinal studies employing standardized assessment protocols are needed to determine whether these changes can reliably predict disease progression.

## 1. Introduction

Dementia exerts a multifaceted impact, placing considerable burden not only on affected individuals but also on their families and society at large. With the global prevalence of dementia projected to triple by 2050—reaching an estimated 150 million cases worldwide [1]—the timely identification of individuals at increased risk of progression across the AD continuum has become a major public health priority. In the absence of disease-modifying therapies capable of preventing or reversing AD, increasing emphasis has been placed on identifying non-invasive behavioral and cognitive indicators that may improve risk stratification and facilitate timely intervention [2].

Within the preclinical spectrum of AD, distinct stages of cognitive decline can be delineated, which are considered transitional throughout the disease trajectory. Subjective cognitive decline (SCD) refers to the persistent self-experienced perception of cognitive deficits relative to a previous level of functioning, typically expressed in the form of complaints, in the absence of objectively measurable impairments due to the subtle nature of the deficits [3,4]. SCD has been associated with an increased risk of progression to later stages of cognitive decline and dementia [4,5]. Moreover, individuals with SCD who exhibit increased amyloid-β deposition appear to be at heightened risk for subsequent objective cognitive decline [6,7,8]. In addition, increased neurodegeneration has been documented in this population, suggesting that SCD may already reflect early pathological processes underlying AD [9].

The prevailing conceptualization of SCD as a marker of preclinical AD posits that these individuals generally retain, and may even exhibit heightened, awareness of subtle cognitive changes that are not yet captured by standardized testing. This preserved metacognitive insight is thought to distinguish SCD from later stages of impairment, in which awareness progressively deteriorates as the underlying disease burden increases and objective deficits become measurable (e.g., [3,10]). At the subsequent stage of the continuum, Mild Cognitive Impairment (MCI) represents the transitional stage between normal ageing and dementia and is commonly classified into amnestic (aMCI) and non-amnestic (naMCI) subtypes according to the predominant cognitive domain affected. Among these, aMCI is consistently associated with a substantially higher risk of progression to Alzheimer’s disease dementia, with Zuliani et al. [11] reporting a 135% greater likelihood of conversion compared with naMCI. Overall, recent evidence estimates that approximately one-third of individuals with MCI progress to AD dementia, with the risk further increasing in the presence of adverse cognitive profiles, genetic susceptibility, biomarker abnormalities, and functional decline [12,13].

However, evidence from a recent systematic review and meta-analysis by Zhao et al. [14] indicates that the course of MCI is not unidirectional, with a pooled reversion rate to normal cognition of 31%, highlighting the potential for some individuals to regain prior cognitive levels and underscoring the importance of early identification and timely interventions. This raises a twofold question. To what extent do individuals experiencing subjective or objective cognitive decline remain aware of both their limitations and preserved abilities, thereby enabling compensatory strategies? And to what extent do the underlying neurobiological mechanisms support the preservation of this capacity despite progressive AD pathology?

Within this context, self-awareness has emerged as a central metacognitive construct for understanding adaptation to cognitive decline. Intact self-awareness refers to an individual’s ability to accurately evaluate their performance in a given domain, reflecting the correspondence between subjective appraisal and objective performance [15]. Conversely, impaired self-awareness is commonly conceptualized as a deficit in metacognitive monitoring [16,17,18]. To explain the cognitive mechanisms underlying the above processes, The Levels of Awareness Model [19] conceptualizes awareness as a hierarchical process involving information registration, performance monitoring, evaluative judgment, and metacognitive self-representation. Similarly, the Cognitive Awareness Model [20,21] proposes that self-awareness depends on the continuous comparison of incoming information with stored self-representations. When this updating process is disrupted, individuals may continue to rely on outdated representations of their cognitive abilities despite accumulating objective evidence of decline, resulting in impaired awareness.

Although changes in self-awareness do not follow a linear course, exhibiting considerable fluctuations across the cognitive decline continuum as well as substantial inter-individual variability, evidence consistently suggests that the discrepancy between subjective and objective evaluations tends to increase as cognitive impairment progresses [16,22]. However, in the early stages of the cognitive decline continuum, the discrepancy between subjective and objective evaluations is often negative. Specifically, individuals with SCD tend to report greater cognitive difficulties despite the absence of objective impairments, exhibiting a distorted form of self-awareness characterized by an overestimation of their difficulties—a condition often referred to as cognitive dysgnosia, which appears to be driven predominantly by affective rather than cognitive factors [4,23,24,25]. As cognitive impairment progresses, this discrepancy reverses, with individuals reporting higher levels of cognitive ability than those objectively demonstrated [26,27]. Already at the stage of MCI, individuals often rate their cognitive performance as higher than what is measured psychometrically or compared to their age-matched peers [28,29]. Even when some degree of metacognitive awareness is preserved, they tend to underestimate the severity of their deficits, as reflected by inflated confidence and inaccurate performance judgments [30]. As cognitive decline advances into the clinical stages of AD, distorted self-awareness may gradually evolve into anosognosia, reflecting the progressive deterioration of metacognitive accuracy and self-monitoring as neurodegenerative changes increasingly compromise compensatory mechanisms [26,31].

Building upon this conceptualization, a dynamic, non-linear model of self-awareness following an inverted U-shaped trajectory across the AD continuum has been proposed [23]. According to this model, self-awareness is initially characterized by heightened awareness during SCD stage, with subjective cognitive complaints representing its primary behavioral manifestation, before progressively declining as neurodegeneration advances, ultimately leading to anosognosia. Importantly, subjective cognitive complaints have been associated with an increased risk of objective cognitive decline and future progression to dementia, particularly in the presence of AD biomarkers [32,33,34]. However, although the transition from preserved awareness to anosognosia has been relatively well characterized in the later stages of the disease, the temporal dynamics underlying the shift from heightened awareness to its gradual decline remain insufficiently understood. Consequently, it is still unclear whether fluctuations in self-awareness may provide clinically meaningful information regarding progression across the AD continuum, particularly during its preclinical stages. Addressing this gap constitutes a central aim of the present review, which seeks to synthesize current evidence on the cognitive and neurobiological correlates of self-awareness across the AD continuum.

### Objectives

Specifically, the primary objective of this systematic review is to provide a comprehensive synthesis of the current evidence regarding the cognitive and neurobiological correlates of self-awareness across the AD continuum. The review is guided by research questions such as: “How does self-awareness fluctuate across the different stages of the AD continuum? Which cognitive functions and neurobiological markers are associated with these changes? To what extent may variations in self-awareness provide clinically meaningful information regarding progression across the AD continuum?” Following the PRISMA guidelines, this systematic review critically synthesizes studies published between 2016 and 2026, integrating current evidence on the cognitive and neurobiological mechanisms underlying self-awareness. Through a comprehensive evaluation of the available literature, the review aims to clarify the stage-dependent trajectories of self-awareness and examine its potential contribution to the identification of individuals at increased risk of progression across the AD continuum.

## 2. Materials and Methods

### 2.1. Search Strategy

The systematic review was conducted in accordance with the Preferred Reporting Items for Systematic Reviews and Meta-Analyses (PRISMA) guidelines for the reporting of systematic reviews and meta-analyses [35]. The completed PRISMA 2020 Checklist is provided in the Supplementary Materials. A systematic literature search was conducted in July 2026 in PubMed, Scopus, ScienceDirect, and Web of Science. The search strategy was structured around three conceptual domains: (a) Alzheimer’s disease-related terms, (b) populations across the Alzheimer’s disease continuum, and (c) self-awareness-related constructs. Representative search terms included Alzheimer’s disease, subjective cognitive decline, mild cognitive impairment, self-awareness, anosognosia, metacognitive awareness, and awareness of deficits, with database-specific adaptations applied where necessary. Studies examining the cognitive and neurobiological correlates of self-awareness were subsequently identified during the screening process according to the predefined eligibility criteria.

The search was restricted to peer-reviewed articles published in English between January 2016 and July 2026. Where available, filters for human studies and original research articles were applied. The review protocol was retrospectively registered with the Open Science Framework (OSF) (https://doi.org/10.17605/OSF.IO/V37CS, accessed on 5 August 2026). No amendments were made to the registered protocol following registration, and all stages of the review were conducted in accordance with the prespecified methodological procedures. A detailed description of the search strategy for each database is provided in Table 1.

### 2.2. Eligibility Criteria

#### 2.2.1. Inclusion Criteria

Inclusion criteria for the review required that studies (1) examine self-awareness as the primary construct of interest, operationalized through discrepancy-based approaches comparing subjective evaluations with objective cognitive performance, informant ratings, or performance-based measures in relation to (2) the cognitive and/or neurobiological correlates of self-awareness across the AD continuum. More specifically, eligible studies involved (3) cognitively unimpaired older adults, individuals with SCD, and MCI and/or AD. Cognitively unimpaired older adults were included either as reference populations or as individuals within the preclinical AD spectrum identified through AD biomarkers. (4) Only primary empirical studies published in peer-reviewed journals were included to ensure the incorporation of original data and analyses. (5) Eligible studies were required to be published in English between January 2016 and July 2026.

#### 2.2.2. Exclusion Criteria

Exclusion criteria were defined as follows: (1) studies that did not examine self-awareness as the primary construct of interest or assessed self-awareness exclusively through subjective cognitive complaints or informant ratings, without a discrepancy-based measure; (2) studies investigating populations outside the AD continuum were excluded; (3) studies that did not examine the cognitive and/or neurobiological correlates of self-awareness were excluded; and (4) review articles, meta-analyses, editorials, conference abstracts, dissertations, preprints, and book chapters were excluded due to the absence of primary empirical data, ensuring that only original research studies were included in the synthesis.

### 2.3. Study Selection

**Figure 1:**
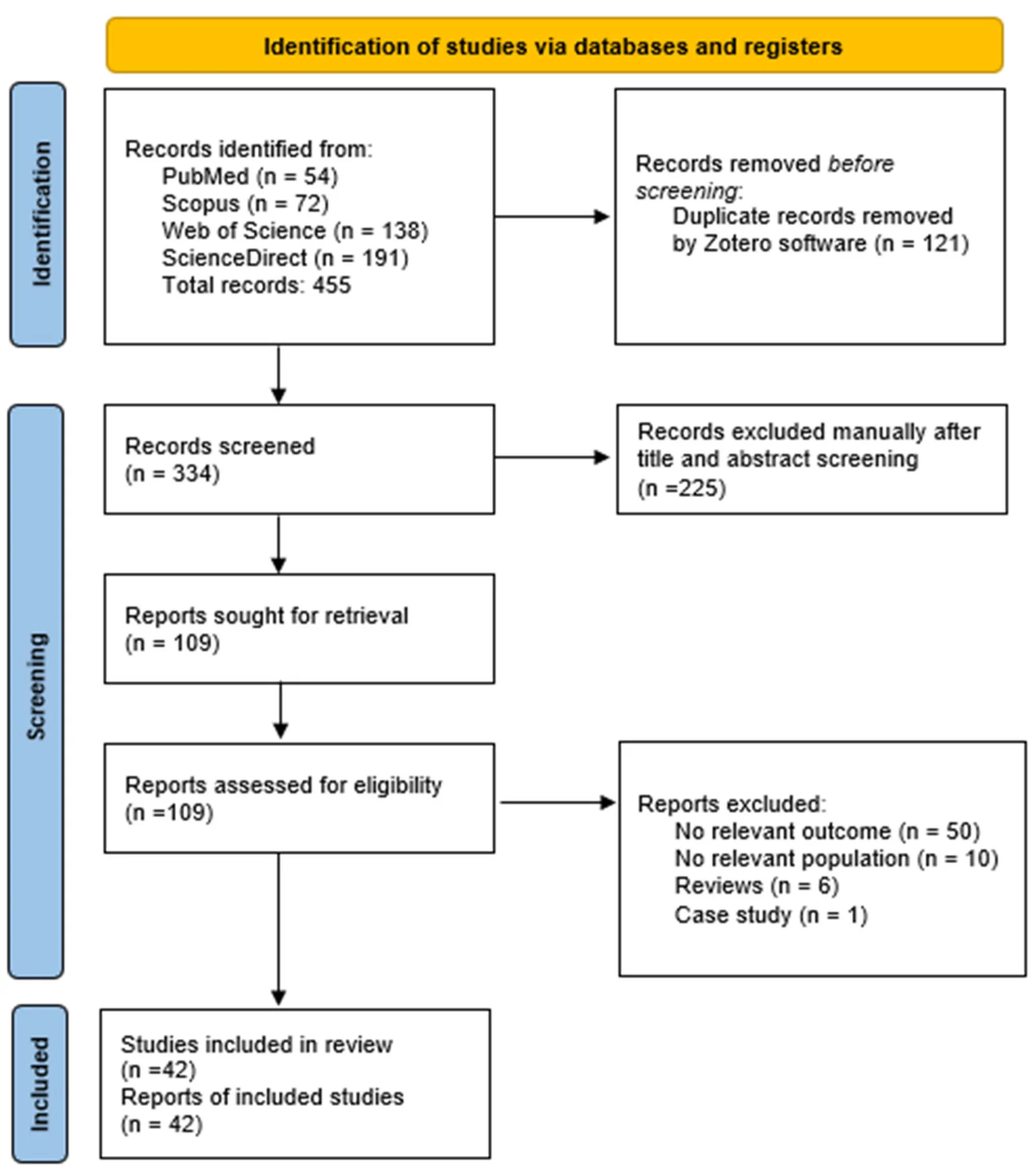
PRISMA 2020 flow chart for the selection of articles included in the systematic review.

The online database search yielded 455 potential studies. Automation tools were used in the selection process to enhance efficiency. Zotero software (version 9.0.6, 64-bit) was used to identify and remove duplicate records. Duplicate removal reduced this number to 334 records, which underwent title and abstract screening by two independent reviewers according to the predefined eligibility criteria. Following this initial screening stage, 225 articles were excluded (Supplementary Table S1) and 109 studies were retained for full-text assessment. Full-text articles were independently evaluated for eligibility by the same reviewers, and any disagreements regarding study inclusion were resolved through discussion and, when necessary, consultation with a third reviewer. Of the 109 full-text articles assessed for eligibility, 67 articles were excluded because they did not satisfy one or more of the predefined eligibility criteria, including ineligible study populations, the absence of discrepancy-based measures of self-awareness, lack of cognitive and/or neurobiological outcomes, or publication types not meeting the inclusion criteria (Supplementary Table S2). Consequently, 42 studies were included in the final qualitative synthesis. The methodological quality of the included studies was subsequently assessed using the Joanna Briggs Institute (JBI) Critical Appraisal Checklists, according to the design of each included study [36]. A detailed overview of the study selection process is presented in the PRISMA flow diagram (Figure 1).

### 2.4. Data Synthesis

A qualitative synthesis was performed to integrate the findings of the included studies (see Table 2). To enhance the clarity and coherence of the results, the data were organized into three thematic sections, structured based on the overarching aims of the present review. These sections concern: (a) the stage-dependent trajectories of self-awareness across the AD continuum, (b) the cognitive correlates associated with these changes, and (c) the corresponding neurobiological correlates, focusing on neuroimaging findings associated with alterations in self-awareness across disease stages. This structure was also adopted for the presentation of the results. Studies included in Table 2 were organized by study design, with longitudinal studies presented first, followed by cross-sectional studies. In addition to the thematic synthesis, descriptive study characteristics, including sample characteristics, study design, assessment methods, and principal findings, were systematically extracted and summarized. 

## 3. Results

### 3.1. The Included Papers

This review included 42 studies investigating the variability of self-awareness within the spectrum of AD. The included studies examined the stage-dependent trajectories of self-awareness and their associations with cognitive performance, AD biomarkers, and neuroimaging findings, with the aim of clarifying the cognitive and neurobiological mechanisms underlying changes in self-awareness across disease progression. Overall, outlining the interactions among self-awareness, cognitive and neuroanatomical indices may contribute to a deeper understanding of the mechanisms underlying changes in self-awareness across the AD continuum and their potential clinical utility. The literature search and selection process, adhering to PRISMA 2020 [35], are depicted in Figure 1. Table 1 provides a detailed summary of the included studies, including sample sizes, study designs, objectives, measures and key findings.

### 3.2. Characteristics of the Included Studies

Across the 42 studies included in this review, self-awareness emerged as a dynamic construct that varied across the AD continuum, associated with both cognitive functioning and neurobiological alterations. All retrieved articles were written in English. Although considerable methodological heterogeneity was observed regarding study design, participant characteristics, and assessment methods, the available evidence collectively supports stage-dependent changes in self-awareness. While cross-sectional studies (*n* = 28) primarily characterized self-awareness at different stages of cognitive decline, longitudinal studies (*n* = 14), although fewer in number, provided important insights into its temporal evolution and its relationship with subsequent cognitive decline and disease progression. To facilitate the interpretation of the narrative synthesis and provide an integrated overview of the principal findings across the AD continuum, the main patterns identified in the included studies are summarized in Table 3.

The included studies represented participants across the AD continuum, with most investigations including mixed cohorts spanning multiple disease stages. Nevertheless, participants with MCI and AD were represented more frequently. This reflects the current emphasis of the literature on characterizing changes in self-awareness during the transition from prodromal to clinical stages of the disease. In contrast, comparatively fewer studies investigated self-awareness in participants with SCD in isolation, highlighting the relatively limited evidence available for the earliest stages of the disease continuum.

Across studies, self-awareness was predominantly assessed using discrepancy-based approaches, with the Everyday Cognition questionnaire (ECog [79]) being the most frequently employed instrument. Other commonly used measures included the Anosognosia Questionnaire—Dementia (AQ-D [80]), the Memory Awareness Rating Scale (MARS [81]), the Cognitive Difficulties Scale (CDS [82]), and Feeling-of-Knowing (FOK [83]) paradigms, depending on the objectives of each study. Cognitive functioning was most frequently assessed using global cognitive screening measures, particularly the Mini-Mental State Examination (MMSE [84]) and the Montreal Cognitive Assessment (MoCA [85]) complemented by domain-specific neuropsychological tests targeting episodic memory and executive functioning.

Biological measures were incorporated in a substantial proportion of the included studies, with more than half integrating AD biomarkers and/or neuroimaging techniques to investigate the neural substrates associated with changes in self-awareness. Neurobiological investigations predominantly employed structural MRI, resting-state fMRI, and amyloid PET, while several studies also incorporated cerebrospinal fluid biomarkers to further characterize AD pathology.

The methodological quality assessment indicated that the included studies generally demonstrated acceptable methodological quality. While all cross-sectional studies fulfilled the appraisal criteria, only minor methodological limitations were identified among some longitudinal studies, primarily related to follow-up completeness and the management of incomplete follow-up. These findings provide additional context for the interpretation of the evidence presented in this review, although the conclusions should be interpreted considering the methodological heterogeneity of the included studies. A detailed overview of the characteristics of the included studies is presented in Table 2.

### 3.3. Results of Synthesis

#### 3.3.1. Stage-Dependent Trajectories of Self-Awareness Across the AD Continuum

Cross-sectional studies collectively demonstrate that self-awareness varies across the AD, although the available evidence does not support a uniform pattern across disease stages, but rather, self-awareness appears to follow a dynamic and, in several cases, non-linear trajectory, with the greatest variability observed during the preclinical phases of AD. While several studies reported heightened awareness and/or increased concern among cognitively unimpaired older adults and individuals with SCD [54,60,67,69,70], others identified subtle reductions in awareness among biomarker-positive individuals despite preserved objective cognitive performance [51,71,75,78]. Overall, these findings indicate that alterations in self-awareness may already be present during the preclinical stages of AD, although their direction appears to vary across individuals and study populations.

Beginning with the studies supporting the notion of heightened awareness during the preclinical stages of AD, Verfaillie et al. [60] reported that greater cortical amyloid burden among SCD was associated with increased concern regarding memory decline and heightened awareness of cognitive changes, whereas amyloid burden was unrelated to the overall severity of subjective cognitive complaints. Specifically, greater amyloid deposition nearly doubled the likelihood of expressing concern about memory decline (OR = 1.76, 95% CI: 1.07–2.90). Similarly, Vannini et al. [54] found that cognitively unimpaired amyloid-positive individuals demonstrated greater memory awareness than amyloid-negative participants, the presence of a hyperawareness profile that differentiated cognitively unimpaired individuals with underlying amyloid pathology from those without amyloid burden. Furthermore, consistent findings were reported by Gagliardi and Vannini [70], who described increased awareness during the cognitively unimpaired stage, followed by progressive reductions across the AD continuum. Likewise, Kuhn et al. [67] identified heightened concern during the earliest disease stages, preceding the subsequent decline in awareness. Notably, this pattern was observed in individuals with preserved global cognitive functioning (MMSE > 28), indicating that increased awareness may already be detectable before the onset of measurable cognitive impairment, suggesting a heightened sensitivity to early neurobiological alterations. Using participant–informant discrepancy measures, Cacciamani et al. [69] further observed that amyloid-positive cognitively unimpaired individuals reported greater memory and language difficulties than their study partners, while maintaining intact objective cognitive performance.

In contrast, reduced awareness even before clinically overt cognitive impairment became apparent has been additionally described. Cacciamani et al. [51] demonstrated that subjective cognitive complaints alone were not associated with cognitive performance or AD biomarkers. Instead, individuals presenting reduced awareness, reflected by reporting fewer cognitive difficulties than their informants, exhibited greater amyloid burden and an Alzheimer’s disease-related hypometabolism despite preserved cognition and functional performance. Similarly, Li et al. [71] found progressively increasing overconfidence among amyloid-positive individuals, with awareness declining across successive stages from SCD to aMCI and AD. López-Martos et al. [75] further described a non-linear relationship between awareness and amyloid pathology, demonstrating that metamemory awareness initially increased towards the edge of hyperawareness during the earliest phases of amyloid accumulation before progressively declining after reaching a threshold of amyloid positivity. Comparable findings were reported by Furuya et al. [78], who showed that reduced participant–study partner discrepancy independently predicted amyloid PET positivity among cognitively unimpaired individuals. More specifically, greater anosognosia was found to be independently associated with amyloid PET positivity (OR = 0.925 per one-point increase in ΔCFI), even before dementia arose, supporting ΔCFI as a clinically useful indicator of preclinical and prodromal AD.

Taken together, cross-sectional findings indicate that awareness changes may already be detectable during the preclinical stages of AD. While several investigations reported heightened awareness or increased concern among cognitively unimpaired and SCD participants, others identified subtle reductions in awareness despite preserved objective cognition, particularly among individuals with evidence of underlying AD pathology.

Findings concerning individuals with MCI were considerably more heterogeneous than those reported for cognitively unimpaired participants or patients with AD dementia. Several studies suggested that awareness remains relatively preserved in at least a proportion of individuals with MCI. For instance, Chi et al. [65] reported preserved memory self-awareness and metacognitive monitoring among participants with SCD and aMCI, whereas impaired monitoring was primarily observed in naMCI. Likewise, Cacciamani et al. [69] identified an intermediate awareness profile in aMCI, positioned between the heightened awareness observed in amyloid-positive cognitively unimpaired individuals and the pronounced anosognosia characterizing AD dementia. Benke and Karagiannis [77] similarly found that overestimation of memory performance was relatively uncommon among participants with MCI compared with patients with AD, suggesting that individuals with MCI largely retained the ability to accurately evaluate their own memory performance despite emerging cognitive deficits.

Conversely, several investigations reported measurable impairments in awareness already during the MCI stage. Ryals et al. [58] demonstrated impaired retrospective awareness of verbal and visuospatial memory despite relatively preserved online monitoring processes, whereas Vannini et al. [55] reported reduced memory awareness among amyloid-positive individuals with aMCI compared with cognitively unimpaired participants. Similarly, as noted above, Li et al. [71] further reported an increased overconfidence among individuals with aMCI, reflected by a larger discrepancy between subjective estimations and objective memory performance.

In contrast to the heterogeneous findings observed in MCI, studies involving AD dementia consistently demonstrated substantial reductions in self-awareness. Across the included literature, anosognosia emerged as the predominant awareness profile, accompanied by increasing overestimation of cognitive abilities and reduced correspondence between subjective evaluations and objective performance. Benke and Karagiannis [77] reported that almost half of patients with mild AD overestimated their memory performance and, importantly, failed to revise these judgments following direct performance feedback. Likewise, Cacciamani et al. [69] identified multidomain anosognosia, with the largest participant–informant discrepancies observed for executive functioning. Similar patterns of progressively declining awareness with increasing disease severity were reported by Kuhn et al. [67], Gagliardi and Vannini [70], and Li et al. [71].

Collectively, the cross-sectional evidence indicates that self-awareness exhibits substantial variability during the preclinical and prodromal stages of AD, whereas findings become considerably more consistent following the onset of dementia. While awareness profiles among cognitively unimpaired individuals, SCD, and MCI remain heterogeneous, studies involving AD dementia consistently describe marked reductions in self-awareness and increasing anosognosia.

On the other hand, longitudinal studies investigated changes in self-awareness across the AD continuum, primarily examining its relationship with clinical progression, cognitive decline, and AD pathology over time. Although the studies differed in follow-up duration, participant characteristics, and assessment methods, most evaluated whether alterations in awareness preceded or accompanied disease progression. A consistent finding across several studies was the association between declining awareness and subsequent clinical progression. In the ADNI cohort, Gerretsen et al. [38] demonstrated that greater anosognosia independently predicted conversion from MCI to AD within five years (adjusted OR = 1.64, 95% CI: 1.12–2.40) after adjustment for demographic variables, APOE ε4 status, dementia severity, and baseline cognitive performance. Similarly, Therriault et al. [41] reported that impaired awareness was associated with a 2.86-fold increased risk of progression from amnestic MCI to dementia during a 24-month follow-up. Munro et al. [40] likewise found that reduced awareness at baseline distinguished individuals who subsequently progressed to AD from those who remained clinically stable. Comparable findings were reported by Bastin et al. [44], who followed 44 participants with aMCI over four years and observed conversion to AD in 23 participants (52.3%), with anosognosia being present exclusively among converters. Edmonds et al. [39] further demonstrated that progressive reductions in awareness accompanied worsening cognitive status and predicted subsequent clinical progression, while informant reports increasingly reflected disease progression more accurately than self-reported cognitive complaints.

Furthermore, several longitudinal studies demonstrated that changes in self-awareness closely paralleled the progressive accumulation of AD pathology. Therriault et al. [41] demonstrated that impaired awareness was associated with greater cerebral amyloid burden, lower CSF Aβ42 concentrations, higher total and phosphorylated tau levels, and progressive hypometabolism in AD-vulnerable brain regions. Hanseeuw et al. [43] similarly reported that progressive amyloid accumulation was accompanied by deterioration in memory self-awareness and the emergence of anosognosia. Specifically, amyloid-positive cognitively unimpaired individuals initially exhibited heightened awareness up to approximately 1.6 years before MCI diagnosis, followed by a gradual decline, whereas awareness continued to deteriorate during the MCI stage, with anosognosia emerging approximately 3 years before dementia onset. Notably, low awareness among cognitively unimpaired individuals predicted subsequent progression to MCI, whereas heightened awareness was not associated with future cognitive decline. Similarly, Mimmack et al. [46] further demonstrated that distinguishing unawareness from heightened awareness improved the prediction of subsequent clinical progression, as each one-point decrease in awareness corresponded to an approximately 5.4-fold increase in the hazard of disease progression. Likewise, Wilkins et al. [48] found that impaired awareness, particularly overestimation of everyday cognitive abilities, predicted accelerated neurodegeneration across the AD continuum. More recently, López-Martos et al. [50] reported that reduced awareness of cognitive decline emerged alongside multimodal AD pathology before clinically overt cognitive impairment, extending current preclinical models of AD and placing the transition from heightened to reduced awareness within the earliest stages of subjective cognitive decline.

Complementing these biomarker findings, other longitudinal studies focused on the clinical evolution of awareness, as well as the cognitive and neural mechanisms underlying its progressive decline, which are discussed in greater detail in the following sections. Silva et al. [37] observed a gradual decline in awareness alongside cognitive deterioration, although awareness alone showed limited predictive accuracy for subsequent conversion to AD. Similarly, Cacciamani et al. [49] demonstrated that awareness changes varied across disease stages, with early reductions accompanying amyloid accumulation and neurodegeneration during the prodromal phase and more pronounced impairment emerging in association with posterior cingulate hypometabolism following dementia onset. In addition, Bregman et al. [42] reported that lower awareness, quantified using participant–informant discrepancies, was associated with a declining memory performance, greater AD pathology and a faster clinical progression in MCI. Chen et al. [45] further associated anosognosia in aMCI with disruption of self-referential and memory-related brain networks, accompanied by greater tau pathology, worsening AD biomarkers, and accelerated progression to dementia. Finally, Razafimahatratra et al. [47] associated declining awareness with impaired error awareness and reduced error-related positivity (Pe), linking awareness decline to alterations in neural mechanisms supporting performance monitoring. Taken together, longitudinal studies consistently demonstrated that declining self-awareness accompanies clinical progression and the accumulation of AD pathology across the disease continuum, although the timing and magnitude of awareness changes varied across study populations and methodological approaches.

#### 3.3.2. Cognitive Underpinnings of Self-Awareness’ Fluctuations

To investigate the cognitive substrates underlying preserved or impaired self-awareness, several studies examined the correspondence between subjective evaluations and objective cognitive performance. In many cases, the discrepancy between subjective and objective performance was also used as an operational measure of self-awareness, enabling the identification of distinct cognitive profiles associated with different levels of awareness. Although reduced awareness was generally accompanied by poorer cognitive performance while preserved or heightened awareness was generally observed in the context of relatively intact objective cognition, these associations varied according to both the cognitive domain examined and the method used to operationalize self-awareness.

Several studies pointed out the preserved global cognition as a common substrate among individuals exhibiting heightened awareness during the preclinical stages of AD. Kuhn et al. [67], for example, reported that heightened awareness was primarily observed among cognitively unimpaired individuals with MMSE scores above 27, whereas declining awareness became increasingly evident as global cognitive performance deteriorated beyond that cut-off. Specifically, lower MMSE or MoCA performance accompanied reduced awareness in individuals with aMCI and AD, particularly when awareness was assessed through participant–informant discrepancies or objective–subjective performance differences [38,53,55,77]. Senturk et al. [53] reported that greater anosognosia among individuals with aMCI and early AD was associated with poorer global cognition, while Vannini et al. [55] similarly observed lower MMSE scores among aMCI participants with poorer memory awareness. Consistent with these findings, Benke and Karagiannis [77] found that the overestimation of memory ability in AD was accompanied by lower MMSE scores and reduced everyday functioning, whereas most individuals with MCI retained a comparatively realistic evaluation of their performance.

Longitudinal findings showed a comparable pattern. Increasing participant–informant discrepancy was accompanied by worsening global cognitive status in MCI and was associated with progression toward dementia [37,38,39,41]. Edmonds et al. [39] observed that increasing anosognosia over 24 months was associated with poorer MMSE performance and broader decline across memory, language, and executive-attentional domains. Hanseeuw et al. [43] likewise reported that declining memory awareness occurred alongside clinical and cognitive progression and preceded dementia onset, while Lopez-Martos et al. [50] reported that reduced awareness of cognitive decline, emerging in those developing AD, was associated with greater longitudinal cognitive and functional decline despite preserved baseline cognition, alongside increased AD pathology. However, some studies indicated that awareness-related changes remained associated with subsequent disease progression independently of baseline cognitive performance. Gerretsen et al. [38] found that anosognosia retained an independent association with progression after adjustment for baseline cognitive performance, and Wilkins et al. [48] reported that overestimation of everyday abilities predicted subsequent brain atrophy independently of baseline cognition.

Among the cognitive domains evaluated, episodic memory and related memory-monitoring processes emerged as the cognitive functions most consistently evaluated in relation to different levels of self-awareness and were examined as key cognitive mechanisms underlying preserved or impaired self-awareness. Findings from cognitively unimpaired individuals and participants with SCD suggested that objective episodic memory impairment was not a prerequisite for heightened awareness. Verfaillie et al. [60] reported that individuals exhibiting greater awareness of subtle memory changes retained normal episodic memory performance, whereas the severity of subjective memory complaints alone was unrelated to objective cognition. Similarly, Vannini et al. [54], Kuhn et al. [67], and Cacciamani et al. [69] demonstrated that cognitively unimpaired amyloid-positive individuals exhibited heightened awareness despite preserved objective cognitive performance. Extending these findings, López-Martos et al. [75] showed that episodic metamemory was characterized by heightened awareness during the earliest stages of amyloid accumulation before progressively declining as pathological burden increased, suggesting that alterations in memory-monitoring processes may precede measurable episodic memory impairment.

In contrast, as objective memory deficits became more pronounced, poorer episodic memory was consistently associated with declining awareness. Li et al. [71] demonstrated that increasing overconfidence paralleled the progressive deterioration of memory performance from amyloid-positive SCD through aMCI to AD. Similarly, Ryals et al. [58] found that poorer verbal and visuospatial episodic memory was associated with impaired retrospective awareness, whereas online monitoring remained comparatively preserved. Vannini et al. [55] likewise reported poorer awareness of delayed memory performance among participants with aMCI than cognitively unimpaired controls. Senturk et al. [53] further associated greater anosognosia with poorer verbal learning and episodic recall, while Gagliardi and Vannini [70] demonstrated that episodic memory, but not executive functioning, partially mediated the relationship between amyloid pathology and reduced awareness, particularly among individuals with MCI.

Longitudinal studies further supported the relationship between episodic memory and awareness trajectories. Participants with MCI who subsequently progressed to AD consistently exhibited poorer memory performance alongside reduced awareness at baseline and greater deterioration during follow-up than clinically stable individuals [40,42,44]. More specifically, Bastin et al. [44] showed that converters overestimated both future recognition performance on episodic FOK tasks and their everyday memory abilities, whereas non-converters maintained levels of awareness comparable to those of cognitively healthy controls. Similarly, Bregman et al. [42] reported that poorer objective memory predicted greater decline in awareness and faster clinical deterioration, while Silva et al. [37] observed that worsening memory performance was accompanied by progressively increasing overestimation of memory abilities, although discrepancy measures alone showed limited predictive accuracy.

Executive functioning was also examined in several studies as a potential cognitive substrate supporting accurate self-awareness, although the observed associations were less consistent than those reported for episodic memory. Senturk et al. [53] demonstrated that poorer executive functioning, including deficits in inhibitory control, cognitive flexibility, and set-shifting, was associated with greater anosognosia among individuals with aMCI. Similarly, Benke and Karagiannis [77] reported that executive dysfunction was associated with a reduced ability among patients with AD to accurately evaluate their memory performance. In the multidomain study by Cacciamani et al. [69], the largest participant–informant discrepancies were observed for executive abilities, despite memory complaints remaining the most frequently reported subjective difficulty across diagnostic groups. Longitudinally, Edmonds et al. [39] further showed that progressive reductions in awareness were accompanied by deterioration in executive-attentional functioning alongside decline in other cognitive domains.

Nevertheless, executive functioning did not consistently account for awareness across disease stages. Gagliardi and Vannini [70] demonstrated that episodic memory, rather than executive functioning measured with the Trail Making Test, mediated the association between amyloid pathology and reduced awareness. Similarly, Ryals et al. [58] reported relatively preserved online monitoring despite impaired retrospective awareness of memory performance, indicating that different components of executive or monitoring may be differentially affected. Chi et al. [65] likewise observed preserved monitoring accuracy in SCD and aMCI, whereas poorer delayed-recall metamemory accuracy and greater overconfidence were primarily observed in individuals with naMCI, further highlighting variability in monitoring performance across MCI subtypes.

Experimental paradigms examining performance monitoring provided additional insight into the executive mechanisms underlying impaired self-awareness. Razafimahatratra et al. [47] demonstrated that individuals who subsequently progressed to AD exhibited reduced error-related positivity (Pe), indicating impaired conscious error monitoring despite relatively preserved early error detection (ERN). Bastin et al. [44] similarly reported impaired episodic FOK accuracy among converters, while Benke and Karagiannis [77] showed limited updating of performance judgments following feedback. Collectively, these findings support an association between alterations in performance monitoring and declining self-awareness across the AD continuum.

#### 3.3.3. Neuroimaging Findings Underlying Alterations in Self-Awareness

The gradual deterioration of self-awareness across the continuum of cognitive decline—from cognitively healthy older adults, in whom normative aging exerts subtle effects on metacognitive integrity, to the extensive destabilization that characterizes the dementia stage—is reflected not only in the individual’s cognitive capacity but also in the integrity of the neurobiological mechanisms that support the formation of self-evaluative judgments. The metacognitive discrepancies observed with advancing age, along with the transition from heightened awareness to diminished awareness, appear to be grounded in a progressive disruption of self-referential processing systems, memory retrieval mechanisms, and executive monitoring functions, mirroring the increasing accumulation of pathological proteins that accompanies disease progression.

Among older adults without yet objectively detectable cognitive impairment, neuroimaging evidence suggests that alterations in self-awareness are associated with early functional changes in brain regions vulnerable to AD pathology. Using FDG-PET, Cacciamani et al. [51] showed that individuals exhibiting reduced awareness relative to caregiver reports presented widespread hypometabolism in frontal, temporal, and parietal regions, accompanied by greater cerebral amyloid burden. The absence of significant hippocampal volume differences further suggests that functional rather than structural alterations may predominate during the earliest disease stages. Similarly, Vannini et al. [55] reported hippocampal hypometabolism in cognitively unimpaired amyloid-positive individuals despite preserved objective cognition and heightened awareness of subtle cognitive changes, supporting the presence of early functional alterations preceding overt structural degeneration.

Beyond hippocampal hypometabolism, evidence also suggests that early alterations in self-awareness are accompanied by subtle structural changes within cortical regions supporting self-referential processing and metacognitive monitoring. Li et al. [71] demonstrated that individuals with SCD and confirmed Aβ pathology exhibited reduced cortical thickness in the medial orbitofrontal cortex, medial prefrontal cortex, anterior and posterior cingulate cortices, insula, middle temporal gyrus, and parahippocampal gyrus. These regions include key nodes of the medial cortical structures and the Default Mode Network (DMN), which are implicated in self-monitoring, performance evaluation, and autobiographical memory processing.

Further evidence suggests that early disturbances in self-awareness may be more closely associated with tau pathology than with amyloid deposition. Vannini et al. [59] demonstrated that reduced accuracy in episodic metamemory judgments was associated with increased tau accumulation in medial temporal regions, including the entorhinal cortex, anterior temporal lobe, and superior temporal gyrus, whereas associations between semantic metamemory monitoring and tau deposition in neocortical regions (superior and inferior parietal lobules, angular gyrus, temporal and orbitofrontal cortices) did not remain significant after correction for multiple comparisons. Consistent with these findings, Zhuang et al. [73] reported that lower global self-assessment among cognitively normal older adults was associated with greater tau deposition in the inferior temporal cortex and, among amyloid-positive individuals, in the entorhinal cortex, despite the absence of cortical atrophy. In contrast, performance-based overestimation was associated with widespread cortical atrophy involving the bilateral inferior temporal, parahippocampal, and fusiform gyri, as well as the right superior temporal gyrus, inferior parietal cortex, and anterior cingulate cortex (ACC), but showed no significant association with AD biomarkers.

The transition from heightened self-awareness to progressive anosognosia was further illustrated by Kuhn et al. [67], who demonstrated, across two independent cohorts, a reversal in the neural correlates of subjective cognitive complaints according to cognitive status. Among individuals with SCD and MCI presenting relatively preserved global cognition (MMSE > 27), greater subjective complaints, reflecting heightened self-awareness, were associated with lower glucose metabolism and reduced gray matter volume in regions affected early by AD, including the frontal and temporal cortices, insula, putamen, and superior frontal gyrus. In contrast, among participants with MCI and AD dementia (MMSE < 27), reduced subjective complaints and impaired awareness were associated with hypometabolism and gray matter loss within key nodes of the DMN, including the ACC, precuneus, medial temporal lobe, prefrontal cortex, claustrum, and thalamus. The above regions lie at the core of self-referential processing and support critical aspects of self-monitoring, with their deterioration paralleling reductions in self-awareness.

**Figure 2:**
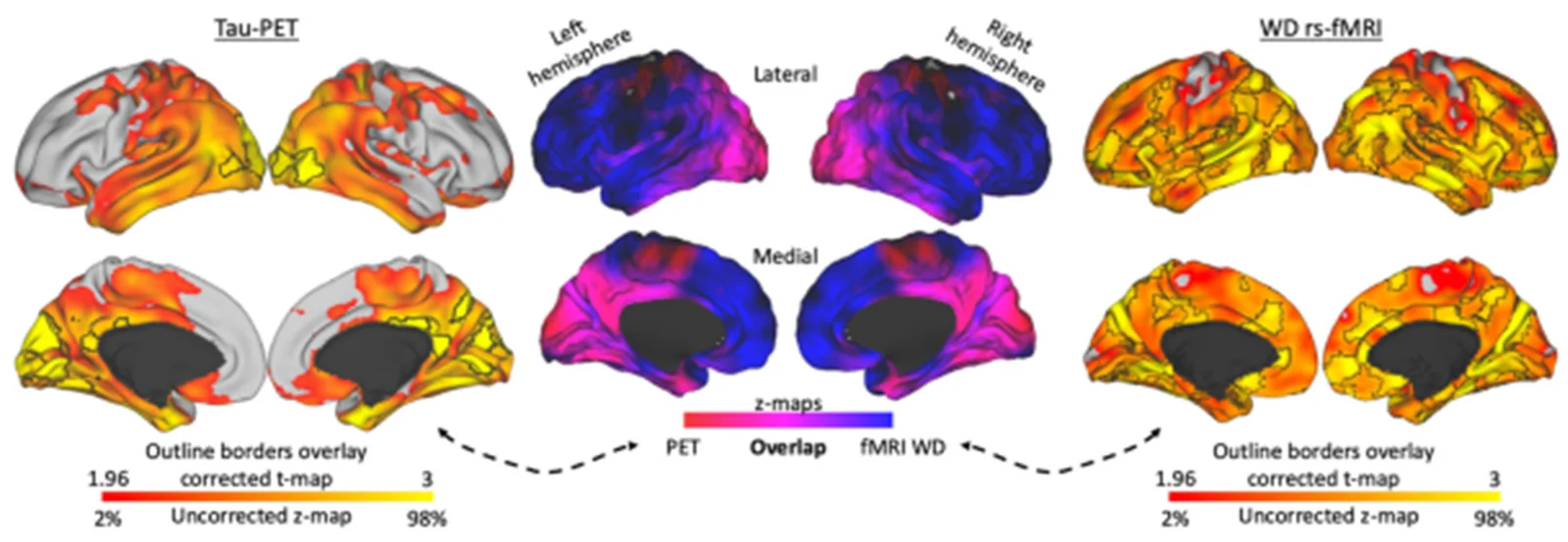
Brain system alterations are associated with unawareness and tau pathology. Individuals with reduced awareness show higher tau deposition and increased functional connectivity compared to aware participants, particularly in posterior cortical regions including occipital, temporal, and parietal areas, suggesting a link between tau accumulation and alterations in large-scale brain networks supporting awareness. Reproduced from Multi-modal Neuroimaging Phenotyping of Mnemonic Anosognosia in the Aging Brain, Bueichekú [74], *Communications Medicine*. Licensed under CC BY 4.0 (http://creativecommons.org/licenses/by/4.0/).

**Figure 3:**
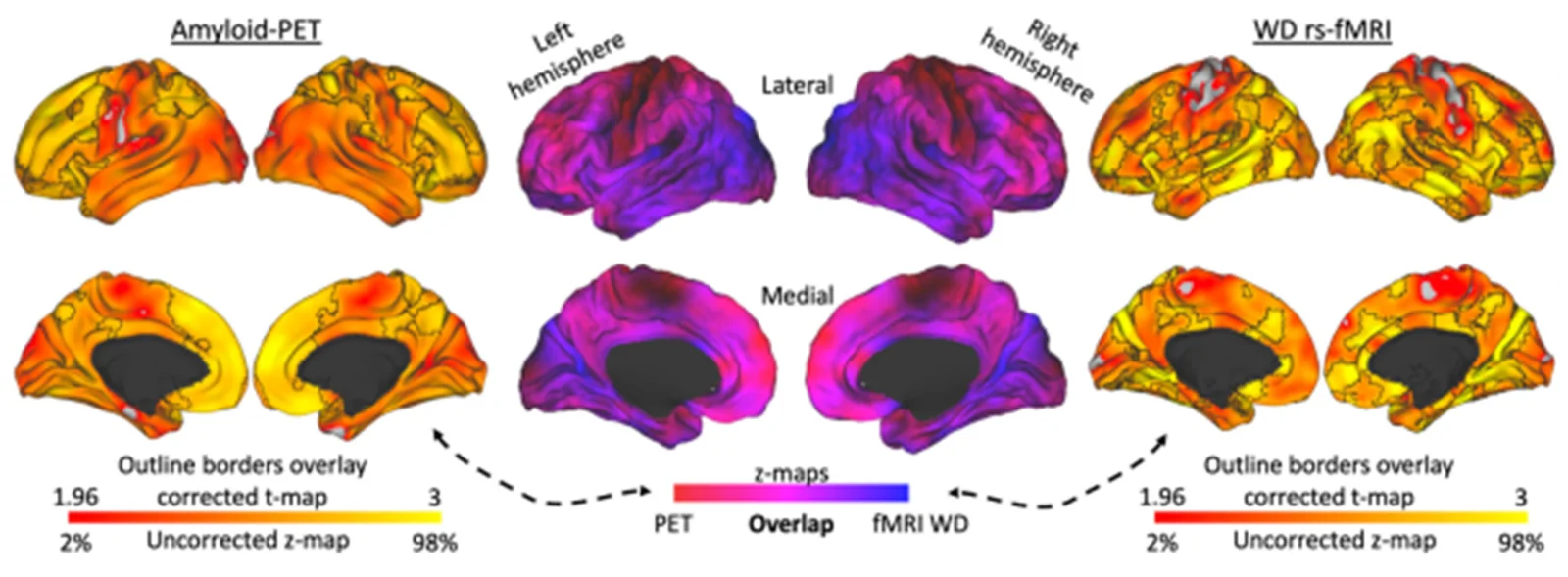
Brain system alterations associated with unawareness and amyloid pathology. Individuals with reduced awareness show increased amyloid accumulation primarily in medial anterior prefrontal regions, extending to dorsolateral prefrontal and superior parietal areas and co-occurring with increased anterior functional connectivity, suggesting an association between amyloid deposition and alterations in prefrontal–parietal networks supporting awareness. Reproduced from Multi-modal Neuroimaging Phenotyping of Mnemonic Anosognosia in the Aging Brain, Bueichekú [74], *Communications Medicine*. Licensed under CC BY 4.0 (http://creativecommons.org/licenses/by/4.0/).

Longitudinal evidence further demonstrated that awareness follows stage-dependent neurobiological alterations across the AD continuum. Cacciamani et al. [49] showed that subtle reductions in awareness emerge during the prodromal stage alongside increasing amyloid burden and early neurodegeneration, whereas more pronounced anosognosia in AD dementia was primarily associated with posterior cingulate hypometabolism. These findings further support the transition from early heightened awareness to progressive impairment of self-awareness as disease pathology advances. Accordingly, Bueichekú et al. [74] demonstrated that reduced self-awareness may emerge before objectively detectable cognitive impairment in association with increased amyloid burden within the anterior DMN, elevated tau pathology in posterior DMN regions, and widespread hyperconnectivity between anterior and posterior midline structures, as well as occipito-temporal connections (Figure 2 and Figure 3). The spatial overlap between pathological protein accumulation and functional hyperconnectivity indicates that early alterations in self-awareness coincide with large-scale network reorganization before the onset of overt cognitive impairment.

**Figure 4:**
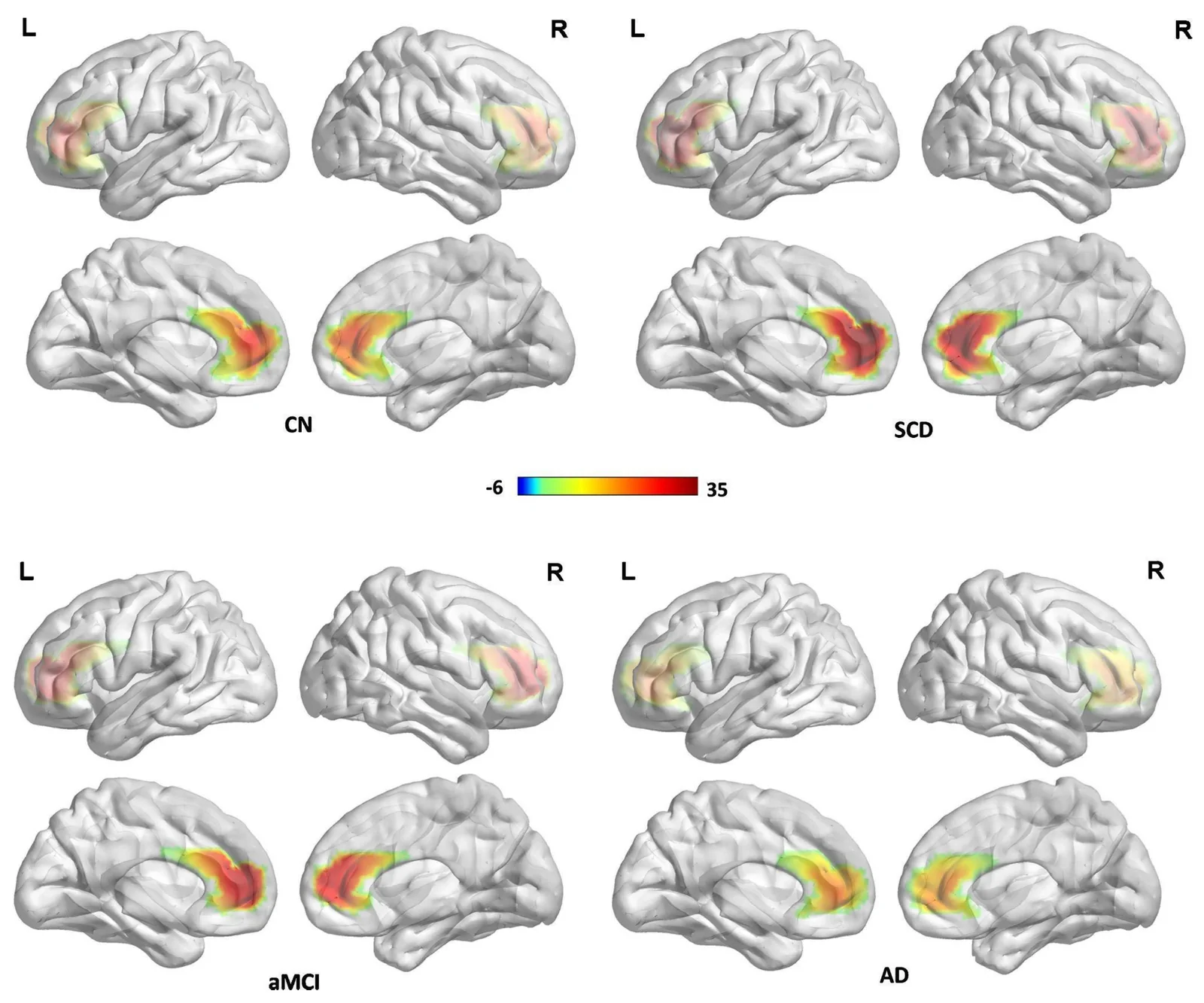
The spatial expression of self-referential networks in CN, SCD, aMCI, and AD groups. Reprinted from Impaired Memory Awareness and Loss Integration in Self-Referential Network Across the Progression of Alzheimer’s Disease Spectrum by Chen et al. [64], *Journal of Alzheimer’s Disease*, 83(1), 111–126. Reprinted with permission.

Similarly, Chen et al. [45,64] demonstrated stage-dependent alterations in functional connectivity within the self-referential processing network, comprising key DMN regions, including the medial ventral prefrontal cortex, orbitofrontal cortex, ACC, middle temporal gyrus, and precuneus. Across successive stages of cognitive decline, connectivity patterns shifted from early compensatory hyperconnectivity to progressive network disruption, with distinct patterns of compensatory connectivity emerging as impairment advanced (Figure 4).

**Figure 5:**
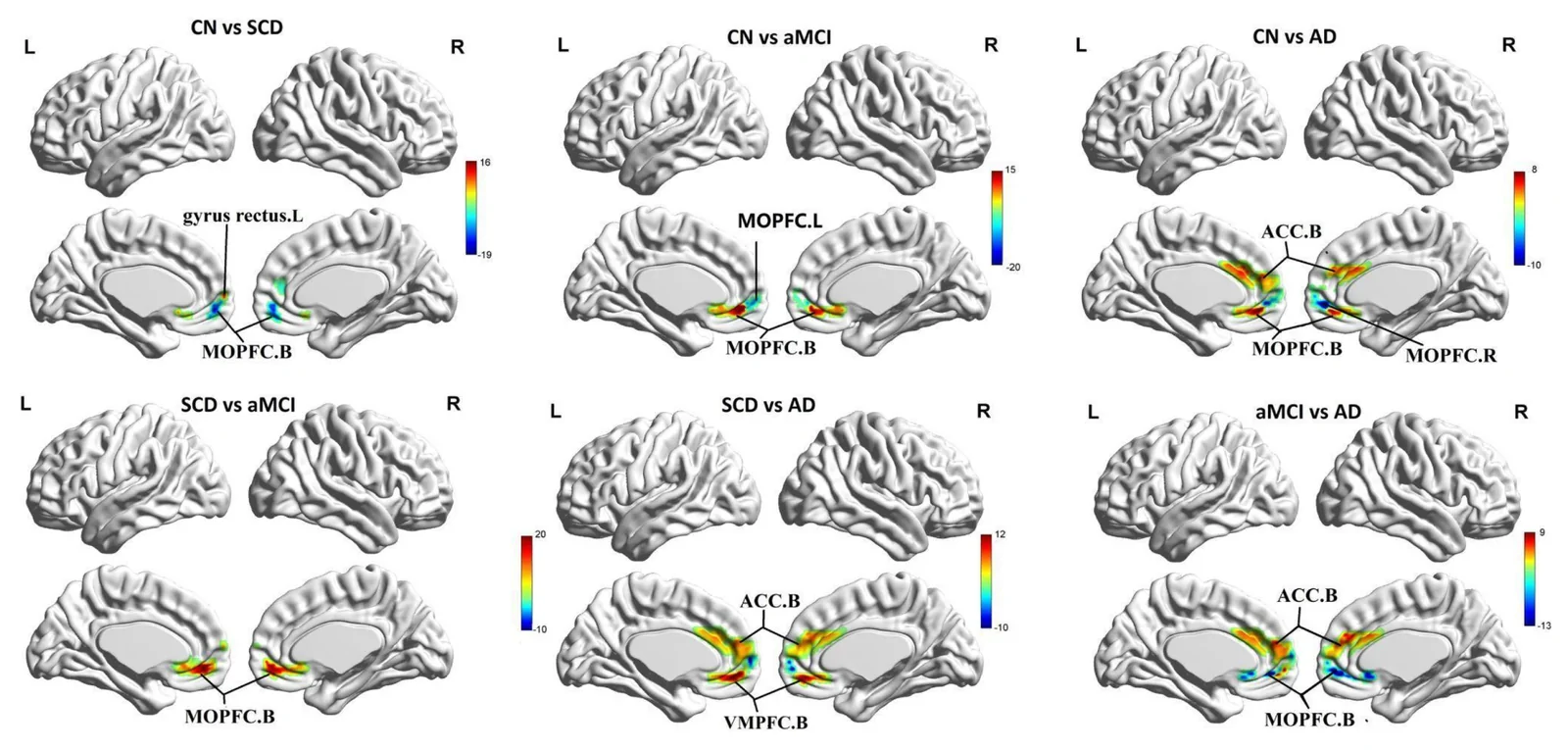
Functional connectivity alterations across the continuum of AD. Reprinted from Impaired Memory Awareness and Loss Integration in Self-Referential Network Across the Progression of Alzheimer’s Disease Spectrum by Chen et al. [64], *Journal of Alzheimer’s Disease*, 83(1), 111–126. Reprinted with permission. Abbreviations: B = bilateral; L = left; R = right; MOPFC = Medial Orbitofrontal Cortex; AA = Anterior Cingulate Cortex; VMPFC = Ventromedial Prefrontal Cortex.

Maximum functional connectivity was observed in individuals with SCD, particularly within the medial orbitofrontal cortex, consistent with the hyperawareness observed at this preclinical stage. Connectivity progressively declined across the AD continuum, with aMCI and AD dementia characterized by widespread hypoconnectivity involving the ventral medial prefrontal cortex, medial orbitofrontal cortex, and ACC (Figure 5; Chen et al. [64]).

Beyond alterations within individual networks, evidence also indicated progressive decoupling between the self-referential network (SRN) and the memory network [45,64]. Among cognitively unimpaired individuals and those with SCD, the two networks exhibited positive functional connectivity, with stronger SRN–memory coupling being associated with greater awareness and better episodic memory performance. As cognitive impairment progressed, this relationship gradually reversed, with individuals with aMCI and AD dementia exhibiting progressive decoupling between self-referential and memory systems, accompanied by declining awareness and poorer episodic memory [64]. Longitudinal analyses further demonstrate distinct connectivity trajectories according to awareness status. Whereas individuals with aMCI without anosognosia showed a progressive reduction in connectivity between the medial orbitofrontal cortex and middle temporal gyrus, as well as between the precuneus and superior temporal gyrus, participants with anosognosia exhibited progressive hyperconnectivity involving the same pathways, together with increased connectivity between the precuneus and cuneus, suggesting a distinct pattern of network reorganization rather than the expected connectivity decline.

Complementing these findings, Chang et al. [63] demonstrated widespread white matter microstructural disruption affecting pathways connecting frontal, thalamic, and interhemispheric regions involved in self-awareness in MCI. At the functional level, Mondragón et al. [68] associated anosognosia in biomarker-positive aMCI with functional alterations centered on the ACC, characterized by increased local connectivity with visual, language, and sensorimotor networks, alongside reduced connectivity within the DMN and cerebellar networks.

The involvement of the ACC in anosognosia further highlights the association of impaired self-awareness with disrupted interactions between the DMN, frontoparietal network (FPN), and salience (SN) networks [72,76]. Valera-Bermejo et al. [72] demonstrated distinct connectivity profiles according to the type of anosognosia. Mnemonic anosognosia was characterized by DMN–FPN hypoconnectivity and reduced ACC and hippocampal connectivity with frontotemporal regions. In contrast, non-mnemonic anosognosia was associated with FPN–SN hyperconnectivity together with enhanced connectivity between the anterior DMN and cerebellum, the ACC and medial prefrontal cortex with the right dorsolateral prefrontal cortex, and the left hippocampus with the insula and caudate nucleus. Multi-domain anosognosia combined DMN–FPN hypoconnectivity with aberrant DMN–SN hyperconnectivity.

Similarly, Tondelli et al. [76] showed that impaired self-awareness in MCI and AD was associated with reduced connectivity within the DMN, particularly involving the posterior cingulate cortex, precuneus, and middle cingulate cortex, alongside increased connectivity within the SN, including the ACC, anterior insula, and basal ganglia. The FPN exhibited reduced connectivity with the posterior cingulate cortex but increased connectivity with the ACC. Notably, functional coupling between the DMN and both the SN and FPN was preserved only in individuals with intact awareness, whereas these interactions were disrupted in patients with anosognosia.

Further evidence of network dysfunction was provided by measures of spontaneous brain activity. Fu et al. [66] reported increased static and dynamic amplitudes of low-frequency fluctuations (sALFF/dALFF) within the ACC, accompanied by reduced activity in the precuneus and other DMN regions. Lower precuneus activity was associated with poorer mnemonic awareness and greater memory impairment, whereas increased anterior cingulate activity was observed during the MCI stage. Complementing these findings, Razafimahatratra et al. [47] demonstrated that declining awareness was accompanied by impaired error awareness together with Pe, whereas ERN remained relatively preserved. Together, these findings link alterations in self-awareness to changes in neural systems involved in self-referential and performance-monitoring processes.

At the biomarker level, Therriault et al. [41] and Chen et al. [45] demonstrated that anosognosia in MCI, particularly the amnestic subtype, was associated with greater amyloid burden within DMN regions and elevated CSF total and phosphorylated tau concentrations involving cortical midline structures, including the medial orbitofrontal cortex, posterior cingulate cortex, and precuneus. Similarly, Vannini et al. [54] and Hanseeuw et al. [43] showed that cognitively impaired individuals without amyloid pathology did not exhibit comparable reductions in self-awareness, supporting an association between anosognosia and underlying AD pathology. Similarly, Lopez-Martos et al. [50] demonstrated that longitudinal changes in awareness closely paralleled the progression of AD pathology. Heightened metamemory awareness during initial amyloid accumulation was followed by progressive deterioration as pathological burden increased, further supporting the dynamic evolution of self-awareness across successive disease stages.

Structural neuroimaging findings consistently implicated the medial temporal lobe in impaired self-awareness. Tondelli et al. [57] demonstrated that reduced awareness was associated with right hippocampal atrophy, involving both anterior and posterior hippocampal regions, regardless of whether awareness was assessed through clinical evaluation, participant–informant discrepancies, or subjective–objective performance discrepancies. This association remained consistent irrespective of age or overall cognitive functioning, supporting the right hippocampus as a robust structural correlate of impaired self-awareness across different assessment approaches.

Beyond structural atrophy, Bastin et al. [61] demonstrated that amyloid-positive individuals with MCI or mild AD and impaired self-awareness exhibited marked reductions in synaptic density involving the bilateral hippocampus, entorhinal cortex, thalamus, and inferior frontal cortex, with the greatest reductions observed in the right anterior hippocampus. In mild AD, synaptic loss extended to additional temporal and frontal regions. Furthermore, reduced self-awareness was associated with lower [18F]UCB-H uptake in the left hippocampus, reflecting lower synaptic density, while reduced synaptic density correlated with poorer global cognitive performance (MMSE) and visual recognition. Together, these findings indicate that hippocampal involvement in impaired self-awareness extends beyond macroscopic atrophy to progressive synaptic disruption.

Longitudinal findings further revealed more extensive neuroimaging abnormalities among individuals who progressed from MCI to AD dementia than among those who remained clinically stable, particularly in the presence of declining self-awareness [44]. Converters showed widespread hypometabolism involving the posterior cingulate cortex, bilateral FPN regions, and the right dorsolateral prefrontal cortex, whereas in non-converters hypometabolism was largely confined to the posterior cingulate cortex. Converters also exhibited more extensive gray matter loss involving the hippocampus, left temporal, parietal and occipital cortices, insula, precuneus, right dorsolateral prefrontal cortex, and left inferior prefrontal cortex. In contrast, non-converters exhibited more restricted atrophy, primarily affecting the posterior hippocampus and left parieto-occipital regions.

However, structural alterations accompanying anosognosia during the MCI stage were not consistently observed across studies, with some findings pointing instead toward functional abnormalities. Senturk et al. [53] found no differences in cortical thickness between anosognosic and non-anosognosic individuals, either in the overall sample or within diagnostic subgroups. Nevertheless, anosognosia was negatively associated with cognitive performance in individuals with aMCI, but not in patients with dementia, suggesting that impaired awareness at early disease stages may occur in the context of functional alterations, even in the absence of detectable cortical thinning.

Consistent with this observation, Vannini et al. [55] demonstrated that reduced awareness in individuals with aMCI was associated with lower glucose metabolism in the posterior medial cortex, particularly the precuneus, as well as in the right mesiotemporal lobe, including the hippocampus and parahippocampal gyrus. Longitudinally, lower baseline awareness predicted further reductions in FDG uptake over 24 months within the posterior cingulate cortex, left basal forebrain, bilateral mesiotemporal regions, and the right lateral temporal cortex [41]. Together, these findings indicate that functional and metabolic alterations associated with impaired awareness may be detectable even when structural changes remain limited or absent.

Functional connectivity analyses by Vannini et al. [55] further demonstrated reduced connectivity between the precuneus and the orbitofrontal cortex, posterior cingulate cortex, and bilateral inferior parietal lobules, with lower awareness consistently associated with weaker connectivity across these regions. Similarly, reduced connectivity between the right hippocampus and the left medial temporal lobe, as well as the right fusiform gyrus, was observed in individuals with anosognosia, further linking impaired self-awareness in aMCI to disrupted connectivity between self-referential and memory-related brain regions.

In AD dementia, impaired self-awareness was associated with more extensive functional and structural brain alterations. Gerretsen et al. [38] demonstrated that reduced awareness was associated with lower glucose metabolism involving the posterior and middle cingulate cortices, precuneus, angular gyrus, and medial orbitofrontal cortex. Similarly, Fujimoto et al. [52] reported that greater anosognosia in patients with mild AD was associated with reduced gray matter volume in the left superior frontal gyrus, further linking impaired self-awareness to structural alterations in frontal regions involved in self-monitoring.

Collectively, these findings indicate increasingly widespread neurobiological alterations associated with anosognosia across the AD continuum, from predominant hippocampal involvement during the MCI stage to increasing involvement of anterior midline regions, particularly the anterior and middle cingulate cortices, in AD dementia [56,57]. Valera-Bermejo et al. [62] further associated anosognosia across both memory and non-memory domains with reduced gray matter volume in the anterior cingulate and fusiform regions, providing additional structural evidence for the involvement of frontal midline regions in impaired self-awareness. Consistent with these findings, Mondragón et al. [68] demonstrated that greater anosognosia was associated with both structural atrophy and hypometabolism of the left ACC. Additional structural alterations involving the left cerebellar vermis, left postcentral gyrus, and right fusiform gyrus were observed in the absence of corresponding metabolic changes, extending the neuroanatomical pattern associated with anosognosia beyond the ACC [56]. Overall, this converging evidence points to the prominent involvement of the ACC, alongside more distributed regional alterations, in impaired self-awareness in dementia.

Within this evolving neurodegenerative context, weakened connectivity between the insula and the hippocampus further supports alterations in neural systems involved in self-referential processing and memory monitoring [38]. Consistent with the connectivity patterns reported by Chen et al. [64] and Mondragón et al. [68], reduced self-awareness in dementia is accompanied by progressive disruption of long-range frontotemporal and frontoparietal connectivity that became more pronounced with advancing cognitive impairment. Reductions in functional connectivity within the DMN, as well as between DMN nodes and medial temporal regions supporting episodic memory, are already evident during the MCI stage and become more pronounced in dementia. Similarly, greater anosognosia is associated with reduced connectivity between the hippocampus and key DMN regions, including the retrosplenial cortex, right posterior inferior parietal lobule, and ventromedial prefrontal cortex, together with disrupted connectivity within the medial temporal subsystem and between this subsystem and the core and dorsomedial DMN subsystems [76]. Overall, these findings reveal an increasingly distributed pattern of altered functional integration across self-referential and memory-related networks as cognitive impairment progresses.

Taken together, the available neuroimaging evidence demonstrates that alterations in self-awareness are accompanied by progressive structural, metabolic, functional, and connectivity changes across the AD continuum. These changes extend from early functional abnormalities within self-referential and memory-related networks during preclinical stages to widespread disruption of large-scale brain networks and medial temporal structures in MCI and AD dementia. Accordingly, the gradual transformation of self-awareness across the continuum of cognitive decline—from cognitively healthy older adults, in whom normative aging exerts subtle effects on metacognitive integrity, to the marked disruption of self-awareness that characterizes the dementia stage—reflects changes not only in cognitive capacity but also in the integrity of the neurobiological mechanisms supporting the formation of self-evaluative judgments. The metacognitive discrepancies observed with advancing age, along with the transition from heightened awareness to diminished awareness, appear to be grounded in a progressive disruption of self-referential processing systems, memory retrieval mechanisms, and executive monitoring functions, mirroring the increasing accumulation of pathological proteins that accompanies disease progression. Overall, the convergence of multimodal neuroimaging findings supports self-awareness as a potentially sensitive marker of disease progression, closely reflecting the evolving neurobiological processes underlying the AD continuum.

## 4. Discussion

The present systematic review sought to synthesize the available current evidence (2016–2026) regarding the cognitive and neurobiological correlates of self-awareness across the AD continuum. Specifically, it aimed to determine (a) how self-awareness changes across successive disease stages, (b) which cognitive and neurobiological mechanisms accompany these changes, and (c) whether alterations in self-awareness provide clinically meaningful information regarding disease progression. Overall, the findings consistently support a dynamic, stage-dependent model of self-awareness, demonstrating that awareness is neither static nor uniformly impaired across the AD continuum. Rather, self-awareness evolves from relatively preserved monitoring in healthy aging to heightened awareness during preclinical stages, followed by progressive destabilization in MCI and ultimately anosognosia in AD [64,67,69,75]. Importantly, these transitions seem to be closely interrelated to complex interaction between cognitive functioning, underlying AD pathology, and the progressive disruption of neural systems responsible for self-monitoring and self-referential processing, suggesting that changes in awareness constitute an active behavioral manifestation of disease progression rather than a simple consequence of global cognitive decline.

At the SCD stage, where persistent subjective complaints constitute a core diagnostic criterion, self-awareness appears to become increasingly dynamic rather than uniformly increased. Although many individuals exhibit hyperawareness, characterized by heightened sensitivity to subtle cognitive changes despite preserved objective performance [60,67], recent biomarker-based studies indicate that early reductions in awareness may already emerge in a subset of cognitively unimpaired, biomarker-positive individuals [74,75]. These findings suggest that hyperawareness is not an obligatory stage but one of several possible trajectories during preclinical AD. Longitudinal evidence further indicates that awareness evolves dynamically, with increased self-monitoring gradually giving way to declining awareness as pathological burden and network dysfunction accumulate [43,46]. Consequently, longitudinal changes in awareness, rather than a single cross-sectional assessment, may provide more clinically meaningful information regarding disease progression, even from the above initial stage of SCD.

MCI appears to represent the critical transitional stage in the evolution of self-awareness, marking the shift from the heightened awareness frequently observed during preclinical AD to the progressive emergence of anosognosia, albeit with considerable interindividual variability. While some individuals retain partial insight into their cognitive difficulties, others exhibit increasing overestimation of abilities and impaired metacognitive updating, reflecting the gradual disruption of monitoring mechanisms [64,70,71]. Longitudinal evidence consistently indicates that declining awareness is associated with increased biomarker burden, accelerated cognitive deterioration, and a two- to threefold higher risk of progression to dementia [38,40,41]. However, awareness alone demonstrates limited sensitivity, and its prognostic value is substantially enhanced when interpreted alongside objective cognitive performance and AD biomarkers [38,39].

By the dementia stage, impaired self-awareness becomes the predominant clinical phenotype, with most patients exhibiting moderate to severe anosognosia that progresses alongside increasing cognitive impairment [38,62]. Patients with AD dementia consistently demonstrate greater discrepancies between subjective evaluations and objective performance, not only in memory but also in other domains, such as instrumental activities of daily living, while insight into selected emotional or social domains may remain relatively preserved during the earlier phases of dementia [86,87]. Nevertheless, the increasing inaccuracy of subjective estimations is further reflected in the stronger correspondence of informant ratings with objective cognitive and functional performance, highlighting the greater clinical utility of informant-based assessments in more advanced disease stages [69,70,87]. On the contrary, in preclinical AD, self-report measures and experimental metamemory paradigms may be more sensitive to subtle cognitive changes that remain undetected by informants, whereas informant-based assessments become progressively more informative during MCI and dementia as self-awareness declines and patient reports increasingly diverge from objective cognitive and functional performance [44,75,88].

Taken together, the available evidence supports conceptualizing self-awareness as a dynamic behavioral marker that follows a non-linear trajectory across the AD continuum rather than a gradual decline parallel to cognitive deterioration. The transition from early hyperawareness to declining insight appears to represent a particularly vulnerable stage, potentially providing clinically meaningful information regarding impending cognitive deterioration [24,43,54]. Although hyperawareness has traditionally been considered the predominant characteristic of the preclinical stages, recent biomarker-based evidence indicates that reduced awareness may already emerge in a subset of cognitively unimpaired or SCD individuals with underlying AD pathology, before clinically detectable cognitive impairment becomes evident [27,74]. Importantly, the association of reduced awareness with multimodal accessed AD pathology suggests that awareness may extend current preclinical AD frameworks by providing an accessible behavioral indicator of ongoing neurobiological changes, thereby complementing established diagnostic approaches.

However, despite the growing body of evidence, the temporal transition from preserved awareness or hyperawareness to declining insight remains insufficiently characterized. The limited availability of longitudinal studies, together with substantial methodological heterogeneity in awareness assessment, restricts understanding of these dynamic changes and limits the comparability of findings across studies. Future longitudinal multimodal investigations employing standardized assessment approaches are therefore essential to establish stage-specific trajectories of self-awareness, clarify their relationship with biomarker evolution, and determine their incremental clinical value for the early identification and monitoring of AD.

Beyond describing the trajectory of self-awareness across the AD continuum and among the cognitive domains examined, episodic memory emerged as the most consistent correlate of reduced awareness across the AD continuum, with poorer memory performance associated with increasing discrepancies between subjective evaluations and objective performance [58,70,71]. However, memory impairment alone does not fully account for anosognosia. Executive functions, including cognitive flexibility, inhibitory control, error monitoring, and metacognitive updating, appear to play an equally important role by enabling individuals to detect performance failures, revise outdated self-beliefs, and incorporate new information into self-evaluative judgments [53,77,89,90]. This interpretation is further supported by experimental metamemory paradigms and recent electrophysiological findings, which suggest that reduced awareness primarily reflects impaired conscious evaluation and updating of performance, rather than a complete inability to detect cognitive errors [50,58]. Taken together, these findings suggest that self-awareness should be conceptualized as a higher-order metacognitive function emerging from the interaction of multiple cognitive systems rather than as a direct consequence of memory decline alone. This multidimensional interpretation also provides a plausible framework for understanding the substantial heterogeneity observed across studies and offers a cognitive basis for the progressive neurobiological alterations underlying anosognosia, discussed below.

The available neuroimaging findings further support the dynamic trajectory of self-awareness across the AD continuum, indicating that changes in awareness parallel the progressive disruption of neural systems supporting self-referential processing, memory, and performance monitoring. Rather than implicating isolated brain regions, the available evidence converges on a network-based model in which early functional alterations gradually evolve into widespread structural and connectivity abnormalities [45,64,72,76]. During cognitively healthy aging and SCD, deviations in awareness appear to reflect early functional reorganization rather than established neurodegenerative changes. Hyperawareness has been associated with subtle amyloid and tau pathology, hippocampal dysfunction, and compensatory alterations within the DMN, suggesting that increased self-monitoring may initially represent an adaptive response to emerging pathological changes [54,55,65,71,75]. However, recent evidence indicates that reduced awareness may already emerge in a subset of biomarker-positive individuals, supporting the existence of heterogeneous neurobiological trajectories during the earliest disease stages [74,75]. As pathology progresses to MCI, awareness decline is accompanied by increasing amyloid and tau burden, hippocampal atrophy, synaptic dysfunction, and progressive disruption of functional connectivity, particularly within the DMN and medial temporal networks [44,57,61,64]. Importantly, functional alterations often precede detectable structural degeneration, suggesting that impaired network communication may constitute one of the earliest neurobiological substrates of anosognosia [41,55].

By the dementia stage, anosognosia reflects widespread network disintegration involving cortical midline structures, the anterior cingulate cortex, hippocampal and medial temporal regions, and disrupted interactions among the DMN, salience, and executive-control networks [56,68,72,76]. Collectively, these findings support the interpretation of anosognosia as a disconnection syndrome resulting from the progressive breakdown of large-scale neural networks responsible for updating self-representations and monitoring ongoing cognitive performance, rather than the dysfunction of a single brain region. Nevertheless, most available evidence derives from cross-sectional studies, highlighting the need for longitudinal multimodal investigations to clarify the temporal relationship between biomarker accumulation, network dysfunction, and the progressive deterioration of self-awareness.

Taken together, the findings of this review suggest that self-awareness should be conceptualized as a dynamic, multidimensional construct that reflects the interaction between cognitive functioning, underlying AD pathology, and the integrity of neural systems supporting self-monitoring. Rather than representing a simple consequence of cognitive decline, changes in awareness appear to capture transitional stages of disease progression, from early compensatory hyperawareness to the emergence of anosognosia as network dysfunction and neurodegeneration advance. This dynamic trajectory highlights self-awareness as a clinically meaningful behavioral marker that may complement neuropsychological assessment and established AD biomarkers, particularly when evaluated longitudinally and interpreted alongside objective cognitive performance and informant reports. Beyond its potential clinical utility as a behavioral marker, these findings also provide a rationale for future research examining whether self-awareness could represent a modifiable target, with interventions aimed at improving metacognitive accuracy potentially supporting emotional adaptation, adaptive self-regulation, and engagement in cognitively meaningful activities aligned with realistically attainable goals.

Nevertheless, the findings of this review should be interpreted considering several limitations. Most of the available evidence derives from cross-sectional studies, limiting conclusions regarding the temporal evolution of self-awareness across the AD continuum. In addition, substantial heterogeneity in the conceptualization and assessment of self-awareness, together with variability in cognitive, neuroimaging, and biomarker methodologies, complicates direct comparisons across studies and limits the generalizability of findings. At the review level, restricting the search to selected electronic databases and English-language publications may have resulted in the omission of relevant studies. Moreover, the qualitative nature of the synthesis did not permit quantitative estimation of effect sizes. Furthermore, although the review protocol was registered in the OSF, the registration was performed retrospectively and, as such, represents a methodological limitation of the present review.

Future research should prioritize longitudinal multimodal studies integrating behavioral measures, experimental metacognitive paradigms, neuroimaging, and fluid biomarkers to clarify the temporal sequence linking pathological burden with changes in self-awareness. Particular emphasis should be placed on identifying the transition from hyperawareness to declining insight, determining whether distinct awareness trajectories characterize different biomarker profiles, and establishing standardized assessment approaches capable of capturing the multidimensional nature of self-awareness. Such advances will be essential for determining the prognostic value of self-awareness and for facilitating its integration into both research protocols and routine clinical assessment.

## 5. Conclusions

Overall, the present review demonstrates that self-awareness constitutes a dynamic and multidimensional behavioral marker that evolves non-linearly across the AD continuum, reflecting the progressive interaction between cognitive decline, AD pathology, and large-scale network dysfunction. By integrating evidence from cognitive, biomarker, and neuroimaging studies, this review highlights that alterations in self-awareness emerge considerably early in the spectrum of cognitive decline and may provide clinically meaningful information regarding disease staging and progression. Importantly, the synthesis suggests that the greatest clinical value of self-awareness lies not in isolated cross-sectional assessments but in its longitudinal evolution and its integration with objective cognitive performance and biological markers.

Future longitudinal multimodal investigations employing standardized awareness measures will be essential to establish stage-specific trajectories, clarify the transition from hyperawareness to anosognosia, and determine the prognostic and clinical utility of self-awareness as a complementary behavioral marker of AD. Ultimately, incorporating awareness assessment into both research protocols and clinical practice may improve early risk stratification, facilitate personalized intervention strategies, and contribute to a more comprehensive understanding not only of the disease progression but also of the individual’s adaptation in this ongoing neurodegenerative process.

## Figures and Tables

**Table 1:** Search terms.

Database	Search Term
PubMed	(“Alzheimer disease” OR “Alzheimer’s disease” OR Alzheimer *) AND (“healthy older adults” OR “cognitively unimpaired” OR “cognitively normal” OR “subjective cognitive decline” OR “SCD” OR “mild cognitive impairment” OR MCI OR “amnestic mild cognitive impairment” OR “aMCI”) AND (“self-awareness” OR “anosognosia” OR “metacognitive awareness” OR “awareness of deficits” OR “awareness of cognitive decline” OR “awareness of cognitive impairment”) All fields Publication Date: 2016–2026
Scopus	TITLE-ABS-KEY ((“Alzheimer disease” OR “Alzheimer’s disease” OR alzheimer *) AND (“cognitively unimpaired” OR “healthy older adults” OR “subjective cognitive decline” OR SCD OR “mild cognitive impairment” OR MCI OR aMCI) AND (“self-awareness” OR anosognosia OR “awareness of cognitive decline” OR “metacognitive awareness” OR “awareness of deficits”)) AND PUBYEAR > 2015 AND PUBYEAR < 2027 AND (LIMIT-TO (LANGUAGE, “English”)) AND (LIMIT TO (SUBJAREA, “MEDI”) OR LIMIT-TO (SUBJAREA, “NEUR”) OR LIMIT-TO (SUBJAREA, “PSYC”)) Limited to: Title-Abstract-Keywords Areas of neuroscience, medicine and dentistry, and psychology Publication Date: 2016–2026
ScienceDirect	“Alzheimer’s disease” AND (“cognitively unimpaired” OR “subjective cognitive decline” OR “mild cognitive impairment” OR “aMCI”) AND (“self-awareness” OR “awareness of cognitive decline” OR “anosognosia” OR “metacognitive awareness”) All fields Areas of neuroscience, Medicine and Dentistry, Psychology Publication date: 2016–2026
WebofScience	((ALL=((“Alzheimer’s disease” OR “Alzheimer disease” OR Alzheimer))) AND ALL=((“cognitively unimpaired” OR “subjective cognitive decline” OR “mild cognitive impairment” OR MCI OR aMCI))) AND ALL=((“self-awareness” OR anosognosia OR “awareness of cognitive decline” OR “awareness of deficits” OR “metacognitive awareness”)) and Article (Document Types) and English (Languages) and Article (Document Types) All fields Publication date: 2016–2026

The asterisk (*) indicates a truncation symbol used to retrieve different word endings and variants of the search term.

**Table 2:** Summary of the included studies examining cognition, biomarkers and neuroimaging data related to self-awareness in AD spectrum.

First Author, Year	Country	Participants	Study Design	Objective	Awareness Construct	Measures	Main Findings
Silva, 2016 [37]	Austria	N = 34 (aMCI = 16, naMCI = 18)	Longitudinal 2-year follow up	To examine longitudinal changes in memory awareness and its association with cognitive decline and AD conversion	Awareness of memory impairment	- FAI discrepancy score (patient–informant) - MMSE, Vienna Neuropsychological Test Battery, VSRT, Boston Naming Test, TMT, WAIS subtests - BDI-II	- Awareness of memory impairment remained relatively stable during the MCI stage but declined with worsening cognitive performance. - Self-awareness showed limited ability to predict conversion from MCI to AD over a two-year follow-up.
Gerretsen, 2017 [38]	USA, Canada	N = 1062 (CN = 372, MCI = 499, AD = 191)	Longitudinal up to 5-year follow up	To investigate the association between anosognosia, cerebral glucose metabolism, and progression from MCI to AD.	Awareness of cognitive decline	- ECog discrepancy score (patient–informant) - ADAS, CDR-Sum of Boxes, MMSE, MOCA, RAVLT, Functional Activities Questionnaire - 18F-FDG PET, APOE ε4 genotype	- Greater anosognosia was associated with reduced glucose metabolism in the posterior cingulate cortex and right angular gyrus. - In participants with MCI, anosognosia independently predicted conversion to AD over a 5-year follow-up.
Edmonds, 2018 [39]	USA	N = 475 [CN = 122, MCI = 353)	Longitudinal 2-year follow-up	To investigate longitudinal changes in awareness of cognitive decline and their associations with cognitive decline, AD biomarkers, and progression to AD.	Awareness of cognitive decline	- ECog discrepancy score (patient–informant) - MMSE, RAVLT, Boston Naming Test, Animal Fluency, TMT-A/B; composite language, memory, and executive/attention scores - CSF Aβ1–42; p-tau/Aβ1–42 ratio; t-tau/Aβ1–42 ratio	- aMCI & mixed MCI showed increasing ECog discrepancy scores, reflecting growing underestimation of deficits. - Increasing unawareness associated with CSF AD biomarker positivity and progression to AD.
Munro, 2018 [40]	USA	N= 33 aMCI	Longitudinal up to 19.1 months follow-up	To investigate the relationship of awareness of and concern about memory performance to progression from MCI to AD.	Awareness of memory performance	- MFQ (residual discrepancy score with objective memory performance) - Composite episodic memory measure, CDR, Seriousness of Forgetting subscale of the MFQ	- Participants who progressed to AD dementia exhibited reduced awareness at baseline, and baseline anosognosia predicted subsequent progression. Anosodiaphoria was not associated with disease progression and appeared to emerge later in the AD continuum.
Therriault, 2018 [41]	USA, Canada	N = 468 (aMCI)	Longitudinal 2-year follow-up	To identify the pathophysiologic mechanisms and clinical significance of anosognosia for cognitive decline in MCI.	Awareness of cognitive decline	- ECog discrepancy score (patient–informant) - MMSE, ADAS-Cog; RAVLT, Category Fluency, TMT-A/B - 18F-Florbetapir PET, 18F-FDG PET, CSF Aβ42, CSF t-tau, CSF p-tau, APOE ε4 status	- Impaired awareness associated with ↓ FDG uptake and ↑ florbetapir uptake in the PCC and predicted progressive hypometabolism within vulnerable regions and DMN dysfunction. - Impaired awareness linked to ~3-fold increased risk of conversion to dementia within 2 years.
Bregman, 2020 [42]	USA, Canada	N = 269 (MCI)	Longitudinal 2-year follow-up	To examine whether the discrepancy between patient and informant can predict MCI prognosis.	Awareness of memory decline	- ECog discrepancy score (patient–informant) - RAVLT, Wechsler Memory Scale—Logical Memory; MMSE - MRI hippocampal volume, CSF Aβ42, CSF p-tau181, CSF p-tau/Aβ42 ratio	- Participants with reduced awareness exhibited poorer memory performance, smaller hippocampal volume, lower CSF Aβ42, higher CSF p-tau181, and greater longitudinal decline over 24 months. - Reduced awareness was associated with greater AD pathology and faster disease progression in MCI.
Hanseeuw, 2020 [43]	USA, Canada	N = 1070 (CN = 360, MCI = 596, AD = 114)	Longitudinal up to 3-year follow up	To characterize change in awareness of memory abilities and its relationship to Aβ burden.	Memory self-awareness	- ECog discrepancy score (patient–informant) - ECog memory domain; MMSE; Logical Memory; CDR - 18F-florbetapir PET; APOE ε4 genotype	- CU individuals showed initial hyperawareness up to ~1.6 years before MCI, followed by gradual decline. - MCI already showing low awareness continued declining (−0.23 discrepant-points/year), reaching anosognosia ~3 years before dementia.
Bastin, 2021 [44]	Belgium	N = 73 (CN = 29, aMCI = 44)	Longitudinal up to 4-year follow-up	To investigate anosognosia in prodromal AD and its neural correlates using FDG-PET and structural MRI.	Awareness of memory functioning	- MARS-MFS discrepancy score (patient–informant), episodic and semantic FOK tasks - DRS; verbal episodic memory; autobiographical memory; Reading Span; Hayling Test; Cognitive Estimation Test; GDS - 18F-FDG PET; structural MRI	- Anosognosia was observed in aMCI patients who later converted to AD. - Reduced awareness was associated with hypometabolism and lower gray matter density in frontal, parietal, and temporal regions.
Chen, 2022 [45]	China	N = 77 (CN = 25, aMCI = 52)	Longitudinal up to 2-year follow up	To investigate the neural correlates of anosognosia in aMCI and its association with AD progression.	Awareness of cognitive decline	- ECog discrepancy score (patient–informant) - MoCA, composite memory score; composite executive function score - 18F-Flortaucipir PET; resting-state fMRI; 18F-FDG PET; CSF Aβ; CSF t-tau; CSF p-tau	- Anosognosia index correlated with AD pathological markers and brain glucose metabolism. - Anosognosia in aMCI is linked to medial OFC–MTG and precuneus–visual cortex circuit dysfunction and predicts faster progression to AD dementia.
Mimmack, 2023 [46]	USA	N = 436 CN	Longitudinal up to 6-year follow up	To investigate the association of self-awareness of memory function with future clinical progression.	Memory awareness	- ECog discrepancy score (patient–informant) - CDR; ECog memory subscale	- Unawareness of memory decline was strongly associated with future clinical progression. - Heightened awareness was not linked to progression risk.
Razafimahatratra, 2023 [47]	France	N = 51 amyloid-positive CN	Longitudinal up to 5-year follow up	To investigate the neural mechanisms underlying anosognosia in preclinical AD using electrophysiological markers of error monitoring.	Awareness of cognitive decline	- HABC-M cognitive subscale discrepancy score (patient–informant) - MMSE; CDR; FCSRT; computerized episodic memory recognition task - 18F-florbetapir PET; EEG	- Participants who progressed to AD developed greater anosognosia and reduced Pe amplitude, whereas ERN remained preserved. - Impaired conscious error monitoring is an early neural mechanism underlying anosognosia in preclinical AD.
Wilkins, 2025 [48]	USA, Canada	N = 794 (CN = 176, SMC = 150, EMCI = 245 LMCI = 148, AD = 75)	Longitudinal up to 2.5-year follow-up	To determine whether impaired insight predicts subsequent brain atrophy across the AD continuum.	Awareness of everyday cognitive functioning	- ECog discrepancy score (patient–informant) - MoCA, MMSE, ECog cognitive domains - Serial 3T structural MRI	- Greater baseline overestimation of cognitive abilities predicted accelerated whole-brain and regional atrophy, particularly in the hippocampus and medial temporal lobe. - Impaired insight independently predicted subsequent neurodegeneration across the AD continuum.
Cacciamani, 2026 [49]	USA, Canada	N = 785 (CN = 263, MCI = 411, AD = 111)	Longitudinal up to ≈ 4.5-year follow-up	To investigate the cross-sectional and longitudinal associations between cognitive awareness and AD biomarkers across the AD continuum.	Awareness of cognitive decline	- ECog discrepancy score (patient–informant) - MMSE. ECog, GDS - 18F-florbetapir PET, 18F-FDG PET, structural MRI	- Declining cognitive awareness showed stage-specific associations with AD biomarkers. - In MCI, impaired awareness was associated with amyloid deposition, cortical atrophy, and posterior cingulate hypometabolism, whereas in AD it was primarily associated with posterior cingulate hypometabolism.
López-Martos, 2026 [50]	Spain	N = 350 CN	Longitudinal up to 3.3-year follow-up	To characterize decreased awareness of cognitive decline and investigate its association with multimodal AD biomarkers in CN.	Awareness of cognitive decline	- Standardized discrepancy between objective memory decline and SCD-Q - FCSRT; MBT; WMS-IV Logical Memory; mPACC; MMSE; WAIS-IV Coding; Semantic Fluency; A-IADL-Q; CDR - CSF biomarkers (Aβ, p-tau, t-tau); plasma p-tau; amyloid PET; tau PET	- Decreased awareness was associated with greater multimodal AD pathology, including CSF, plasma, amyloid PET, and tau PET biomarkers. - Reduced awareness also predicted greater longitudinal cognitive and functional decline despite preserved baseline cognition.
Cacciamani, 2017 [51]	France	N = 318 SCD	Cross-sectional	To investigate whether awareness of cognitive decline better identifies preclinical AD than subjective cognitive complaints.	Awareness of cognitive decline	- HABC-M Cognitive Scale discrepancy score (patient–informant) - Frequency of Forgetting Questionnaire, the INSIGHT Questionnaire of Cognitive Decline, the Assessment of Complaints, the Analogic Scale for Complaints and the AD-related anxiety questionnaire - MMSE, CDR, FCSRT, Rey–Osterrieth Figure, Digit Span, FAB, TMT, Verbal Fluency, composite cognitive scores - Amyloid PET, FDG-PET, structural MRI	- Reduced awareness, rather than subjective cognitive complaints, was associated with greater amyloid burden and AD-related hypometabolism despite preserved cognition. - Awareness-based measures more accurately identified biomarker-positive preclinical AD than subjective cognitive complaints alone.
Fujimoto, 2017 [52]	Japan	N = 49 mild AD	Cross-sectional	To investigate the characteristics and neural correlates of anosognosia.	Memory self-awareness	- Patient–caregiver discrepancy questionnaire (adapted from Squire & Zouzounis) - MMSE, CDR, NPI, GDS - Structural MRI	- Memory-related anosognosia negatively correlated with gray matter volume in the left superior frontal gyrus. - No association observed between anosognosia and medial temporal lobe volume despite typical AD involvement.
Senturk, 2017 [53]	Turkey	N = 47 (aMCI = 26, early AD = 21)	Cross-sectional	To investigate the cognitive and structural correlates of anosognosia in aMCI and early AD.	Anosognosia	- CIRS; AQ-D discrepancy score (patient–informant) - MMSE, CVLT, Digit Ordering Test, Digit Span, Stroop, TMT, Clock Drawing Test, WCST - Structural MRI	- Anosognosia correlated with episodic memory, working memory, and executive functioning in the total sample and aMCI group. - Anosognosia in the early stages of AD anosognosia may reflect functional alterations rather than structural cortical changes.
Vannini, 2017 [54]	USA	N = 297 (CN = 262, MCI =35)	Cross-sectional	To investigate the relationship between memory self-awareness and cerebral amyloid burden.	Memory self-awareness	- MFQ (General Frequency of Forgetting—subjective/objective memory discrepancy score) - MMSE, Logical Memory II (WMS-R), GDS -Amyloid PET (PiB-PET)	- Normal adults harboring Aβ demonstrated heightened awareness. - MCI patients with increased Aβ pathology demonstrated anosognosia. - MCI patients with low Aβ burden have normal insight into their memory functions.
Vannini, 2017 [55]	USA	N = 286 (CN = 251, aMCI = 35)	Cross-sectional	To investigate the metabolic and functional neural correlates of memory anosognosia in prodromal AD.	Memory self-awareness	- MFQ (subjective/objective memory discrepancy score) - MMSE; Logical Memory II (WMS-R); MFQ - FDG-PET; resting-state fMRI	- MCI subjects underestimate their memory deficits. - Anosognosia is related to reduced posterior cingulate and hippocampal metabolism and to a disconnection between regions subserving self-reflection.
Guerrier, 2018 [56]	France	N = 65 (CN = 35, 30 AD)	Cross-sectional	To investigate the structural and metabolic neural correlates of anosognosia in early AD.	Awareness of cognitive decline	- CDS discrepancy score (patient–informant) - FCSRT, FAB, TMT, Digit Span, verbal fluency - Structural MRI, FDG-PET	- Anosognosia associated with atrophy and hypometabolism in the left dorsal anterior cingulate cortex. - No significant association between anosognosia and neuropsychological test performance.
Tondelli, 2018 [57]	Italy	N = 27 (MCI = 15, AD = 12)	Cross-sectional	To compare the neural correlates of anosognosia across three awareness measures in early AD.	Awareness of cognitive decline	- CIRS, AQ-D, Self-appraisal Discrepancy on tests of memory & executive function - MMSE; Babcock Story Recall; RAVLT, Rey–Osterrieth Complex Figure; FAB; Stroop; verbal fluency; executive function tests - Structural MRI	- Greater anosognosia (across CIRS, AQ-D, SADs) associated with increased medial temporal lobe atrophy, including the right hippocampus. - Anosognosia primarily reflects decline in mnemonic processes driven by medial temporal degeneration.
Ryals, 2019 [58]	USA	N = 29 (CN = 15, aMCI = 15)	Cross-sectional	To compare multiple components of memory awareness in individuals with aMCI and healthy older adults.	Memory self-awareness	- Experimental metamemory battery (global predictions, global postdictions, JOL, confidence ratings, modified FOK) - MMSE, Logical Memory I & II, RAVLT, Digit Span, TMT, Category Fluency, Boston Naming Test	- Individuals with aMCI showed impaired retrospective memory awareness and reduced confidence monitoring despite relatively preserved online metamemory processes.
Vannini, 2019 [59]	USA	N = 102 CN	Cross-sectional	To investigate the association between episodic and semantic metamemory and AD pathology in cognitively unimpaired older adults.	Metamemory	- FOK task (episodic and semantic) - MMSE, SRT (delayed recall), Category Fluency Test, GDS - Tau PET, amyloid PET, structural MRI	- Decreased meta-memory processing is related to tauopathy. No association was found with amyloid pathology. - Decreased meta-memory may be a sensitive clinical indicator of AD pathophysiology in the pre-symptomatic phase.
Verfaillie, 2019 [60]	Netherlands	N = 106 SCD	Cross-sectional	To investigate the association between amyloid burden and memory deficit awareness in individuals with SCD.	Memory self-awareness	- CCI discrepancy score (patient–informant) - MMSE, RBMT, RAVLT, CCI, SCF - 18F-florbetapir PET, structural MRI	- Amyloid burden associated with greater memory deficit awareness (hypernosognosia). - Worries about perceived decline may represent an early marker of amyloid pathology, rather than reflecting actual cognitive complaint severity.
Bastin. 2020 [61]	Belgium	N = 43 (CN = 19, MCI = 6, AD = 18)	Cross-sectional	To investigate the relationship between synaptic density and awareness of cognitive and memory functioning.	Awareness of cognitive and memory functioning	- AQ-D, MARS-MFS discrepancy score (patient–informant) - MMSE; DMS-48 - SV2A PET, amyloid PET, structural MRI, FDG-PET	- Reduced synaptic density, particularly in the hippocampus, was associated with greater unawareness of memory deficits.
Valera-Bermejo, 2020 [62]	UK	N = 53 (MCI due to AD, mild AD)	Cross-sectional	To investigate the neuropsychological and structural correlates of domain-specific anosognosia in early AD.	Memory and non-memory awareness	- Measurement of Anosognosia Instrument discrepancy score (patient & informant) - MMSE, Rey–Osterrieth Complex Figure, Raven Progressive Matrices, Stroop, Token Test, Digit Span, WAIS Similarities - Structural MRI (voxel-based morphometry)	- Greater anosognosia was associated with poorer cognitive performance and reduced gray matter volume in frontal and cingulate regions. - Memory anosognosia was primarily associated with episodic and semantic memory dysfunction.
Chang, 2021 [63]	Taiwan	N = 87 (CN = 41, MCI = 46)	Cross-sectional	To investigate the cognitive and white matter correlates of impaired memory awareness in MCI.	Memory self-awareness	- Discrepancy between subjective memory complaints (MCI) and objective episodic memory performance - WMS-III Logical Memory, CVLT-II; Matrix Reasoning, Design Fluency, Digit Span, Stroop, Digit Symbol, Vocabulary, Animal Fluency, CDR, GDS - DSI, structural MRI	- MCI participants with poor memory awareness showed reduced white matter integrity in frontal–subcortical and parietal pathways. - White matter abnormalities remained significant supporting a disconnection syndrome underlying anosognosia.
Chen, 2021 [64]	China	N = 444 (CN = 86, SCD = 156, aMCI = 146, AD = 56)	Cross-sectional	To investigate memory awareness and self-referential network connectivity across the AD continuum.	Memory self-awareness	- ECog discrepancy score (patient–informant) - MMSE, ADNI-Mem, GDS, ECog memory subscale - Resting-state fMRI	Individuals with SCD exhibited hyperawareness and increased self-referential network connectivity, whereas aMCI and AD showed progressive anosognosia accompanied by reduced self-referential network connectivity and disrupted interactions with memory-related networks.
Chi, 2021 [65]	USA	N = 258 (CN = 120, SCD = 82, aMCI = 18, naMCI = 38)	Cross-sectional	To examine differences in memory self-awareness and memory self-monitoring between CU and prodromal dementia groups.	Memory self-awareness	- CCI discrepancy score (patient–informant) - Comprehensive Assessment of Prospective Memory, Brief Visuospatial Memory Test-Revised, GDS	- Memory self-awareness remained largely preserved in individuals with SCD and aMCI, whereas impaired metamemory accuracy and greater overconfidence were observed in naMCI.
Fu, 2021 [66]	China	N = 121 participants (CN = 39, MCI = 82	Cross-sectional	To investigate the functional neural correlates of anosognosia in MCI using resting-state fMRI.	Awareness of cognitive decline	- ECog discrepancy score (patient–informant/memory subscale) - MMSE; Story Recall, RAVLT, Verbal Fluency, TMT A/B; Clock Drawing Test - Resting-state fMRI, structural MRI	- Compared with MCI participants without anosognosia, those with anosognosia showed reduced intrinsic activity in the precuneus and increased activity in the anterior cingulate cortex.
Kuhn, 2021 [67]	France, USA, Canada	N = 904 (CN = 67/147, SCD = 30/84, MCI = 50/369, AD = 36/121)	Cross-sectional	To investigate memory awareness and its association with neurodegeneration across the AD continuum.	Memory self-awareness	- CDS, ECog (objective–subjective memory discrepancy) - RAVLT, MMSE - Structural MRI; FDG-PET; amyloid PET; APOE ε4	- Memory awareness progressively declined across the AD continuum, shifting from heightened awareness in SCD to anosognosia in MCI and AD. - This trajectory was associated with increasing neurodegeneration.
Mondragón, 2021 [68]	Netherlands	N = 121 (CN = 26, aMCI = 78, AD = 17)	Cross-sectional	To investigate the functional neural correlates of anosognosia across the biologically defined AD continuum using the AT(N) framework.	Memory self-awareness	- ECog discrepancy score (patient–informant) - MMSE, MoCA, CDR-SB, ADAS-Cog - Resting-state fMRI, amyloid PET, CSF Aβ42; CSF p-tau181, FDG-PET	- Greater anosognosia in prodromal AD was associated with altered anterior cingulate cortex connectivity, characterized by reduced connectivity with the default mode network and increased connectivity with visual, language, and sensorimotor networks.
Cacciamani, 2022 [69]	USA/Canada	N = 591 (Aβ− CN = 211, Aβ+ CN = 118, Aβ+ aMCI = 191, Aβ+ AD = 71)	Cross-sectional	To compare awareness of cognitive decline across cognitive domains throughout the AD continuum.	Awareness of cognitive decline	- ECog discrepancy score (patient–informant) - MMSE, ADNI memory, language, executive, and visuospatial composite scores - Amyloid PET, CSF Aβ42	- Awareness followed a stage-dependent pattern, with heightened awareness in Aβ+ cognitively normal individuals, relatively preserved awareness in aMCI, and multidomain anosognosia in AD, particularly for executive functioning.
Gagliardi, 2022 [70]	USA/Canada	N = 895 (CN = 362, MCI = 422, AD = 111)	Cross-sectional	To determine whether episodic memory or executive functioning mediates the association between amyloid burden and awareness across the AD continuum.	Memory self-awareness	- ECog discrepancy score (patient–informant) - Logical Memory II, TMT B–A, MMSE - Amyloid PET (AV45)	- Greater amyloid burden was associated with lower awareness across the AD continuum. - Episodic memory, but not executive functioning, partially mediated this association, particularly in MCI.
Li, 2022 [71]	China	N = 697 (CU = 196, SCD = 261, aMCI = 161, AD = 79)	Cross-sectional	To characterize metamemory across the AD continuum and investigate its association with cognition, amyloid and structural brain changes.	Memory self-awareness	- DOC-SD and DOC-LD discrepancy scores during AVLT-H - MMSE, MoCA-B, AVLT-H, Boston Naming Test, Animal Fluency Test - 18F-florbetapir-PET, structural MRI	- Amyloid-positive SCD participants showed subtle metamemory impairment before objective cognitive decline. - Overconfidence increased progressively from SCD to aMCI and AD and was associated with cortical thinning in self-referential brain regions.
Valera-Bermejo, 2022 [72]	UK	N = 53 (MCI = 29, mild AD = 24)	Cross-sectional	To investigate the functional network correlates of memory, non-memory, and global anosognosia in early AD.	Multi-domain anosognosia	- Measurement of Anosognosia Questionnaire discrepancy score (patient–informant) - MMSE - Resting-state fMRI	- Memory anosognosia associated with reduced fronto-temporal connectivity and increased parieto-temporal connectivity. - Total anosognosia scores reflected large-scale network alterations.
Zhuang, 2022 [73]	USA	N = 151 CN	Cross-sectional	To investigate self-appraisal discrepancy and their relationship with cortical thickness and AD biomarkers.	Metacognitive self-appraisal	- Online self-appraisal ratings, appraisal discrepancy score - CVLT, Digit Span; Stroop, Verbal Fluency, Category Fluency, MMSE, GDS - Structural MRI, amyloid PET, tau PET	- Lower self-appraisal was associated with greater tau burden, particularly in individuals with elevated amyloid, whereas overestimation of performance was associated with cortical thinning rather than AD biomarkers.
Bueichekú. 2024 [74]	USA	N = 1048 CN	Cross-sectional	To describe from a multi-modal neuroimaging approach the brain phenotype underlying unawareness in the aging brain.	Memory self-awareness	- MAC-Q, Logical Memory, FCSRT (subjective–objective memory discrepancy) - MMSE - Amyloid PET; tau PET; resting-state fMRI; structural MRI	- Impaired self-referential processing underlying reduced awareness is associated with amyloid/tau accumulation and functional connectivity disruptions in medial frontal and parieto-occipital brain regions.
López-Martos, 2024 [75]	Spain	N = 314 CN	Cross-sectional	To investigate the association between CSF AD biomarkers and episodic memory awareness across the preclinical AD continuum.	Memory self-awareness	- MBT objective–subjective discrepancy, SCD-Q discrepancy score (participant–informant) - MBT, HADS - CSF Aβ42/40; CSF p-tau181; CSF t-tau; APOE ε4	- Episodic memory awareness showed a non-linear association with CSF Aβ42/40, shifting from hypernosognosia to anosognosia as amyloid pathology increased. Individuals classified as unaware decliners exhibited higher CSF p-tau181 levels and poorer metamemory.
Tondelli, 2024 [76]	Italy/UK	N = 60 (MCI = 30, AD = 30)	Cross-sectional	To investigate the association between anosognosia and functional connectivity across the AD continuum.	Awareness of cognitive decline	- AQ-D discrepancy score (patient–informant) - MMSE, MoCA, comprehensive neuropsychological assessment - Resting-state fMRI	- Greater anosognosia was associated with reduced Default Mode Network connectivity, increased Salience Network connectivity, and disrupted Frontoparietal Network interactions. - Large-scale network dysfunction as a neural substrate of impaired awareness.
Benke, 2025 [77]	Austria	N = 228 (CN = 62, MCI = 48, AD = 118)	Cross-sectional	To compare predictive and online memory awareness in AD, MCI, and cognitively normal older adults.	Memory awareness	- Performance-based memory self-ratings (Anosognosia Ratio) - CERAD Neuropsychological Battery, MMSE, DAD, GDS	- Nearly half of the AD group overestimated their memory performance, whereas overestimation was rare in MCI. - Most AD participants failed to update their self-evaluation after objective memory testing.
Furuya, 2025 [78]	USA, Japan	N = 4915 (CN)	Cross-sectional	To investigate whether participant–study partner discrepancy on the CFI predicts amyloid positivity.	Awareness of cognitive decline	- CFI participant–study partner discrepancy (ΔCFI) - CDR, CFI, GDS - Amyloid PET; APOE ε4 genotype	- Both lower CFI-self scores and lower self–informant discrepancy were associated with positive amyloid PET. - Assessing the degree of anosognosia may improve prediction of amyloid PET positivity.

MCI = Mild Cognitive Impairment, aMCI = Amnestic Mild Cognitive Impairment, naMCI = Non-amnestic Mild Cognitive Impairment, AD = Alzheimer’s Disease, FAI = Forgetfulness Assessment Inventory, MMSE = Mini-Mental State Examination, VSRT = Verbal Selective Reminding Test, TMT = Trail Making Test, WAIS = Wechsler Adult Intelligence Scale, BDI-II = Beck Depression Inventory-II, CN = Cognitively Normal, ECog = Everyday Cognition Questionnaire, ADAS = Alzheimer’s Disease Assessment Scale, CDR = Clinical Dementia Rating Scale, MOCA = Montreal Cognitive Assessment, RAVLT = Rey Auditory Verbal Learning Scale, FDG-PET = Fluorodeoxyglucose Positron Emission Tomography, APOE ε4 = Apolipoprotein E epsilon 4 allele, CSF = Cerebrospinal Fluid, Aβ = Amyloid-beta, p-tau = Phosphorylated tau, t-tau = Total tau, MFQ = Memory Functioning Questionnaire, ADAS-Cog = Alzheimer’s Disease Assessment Scale—Cognitive Subscale, Florbetapir PET = [^18^F]florbetapir Positron Emission Tomography, PCC = Posterior Cingulate Cortex, DMN = Default Mode Network, MARS-MFS = Memory Awareness Rating Scale–Memory Functioning Scale, FOK = Feeling of Knowing, JOL = Judgments of Learning, DRS = Mattis Dementia Rating Scale, GDS = Geriatric Depression Scale, OFC = Orbitofrontal Cortex, MTG = Middle Temporal Gyrus, HABC-M = Healthy Aging Brain Care Monitor, FCSRT = Free and Cued Selective Reminding Test, EEG = Electroencephalography, ERN = Error-Related Negativity, Pe = Error Positivity, SMC = Subjective Memory Complaint, EMCI = Early Mild Cognitive Impairment, LMCI = Late Mild Cognitive Impairment, SCD-Q = Subjective Cognitive Decline Questionnaire, MBT = Memory Binding Test, WMS-IV = Wechsler Memory Scale, Fourth Edition, mPACC = Modified Preclinical Alzheimer Cognitive Composite, A-IADL-Q = Amsterdam Instrumental Activities of Daily Living Questionnaire, WAIS-IV = Wechsler Adult Intelligence Scale, Fourth Edition, FAB = Frontal Assessment Battery, DOC-SD = Degree of Confidence—Short Delay, DOC-LD = Degree of Confidence—Long Delay, AVLT-H = Auditory Verbal Learning Test—Huashan version, MoCA-B = Basic Montreal Cognitive Assessment, fMRI = Functional Magnetic Resonance Imaging, CCI = Cognitive Change Index, SCF = Subjective Cognitive Functioning questionnaire, RBMT = Rivermead Behavioural Memory Test, CVLT-II = California Verbal Learning Test—Second Edition, WMS-III = Wechsler Memory Scale—Third Edition, DSI = Diffusion Spectrum Imaging, CDS = Cognitive Difficulties Scale, AQ-D = Anosognosia Questionnaire—Dementia, CIRS = Clinical Insight Rating Scale, MAC-Q = Memory Assessment Clinics Questionnaire, SV2A = Synaptic Vesicle Glycoprotein 2A, DMS-48 = Delayed Matching-to-Sample 48-item Test, CERAD = Consortium to Establish a Registry for Alzheimer’s Disease, DAD = Disability Assessment for Dementia, PiB-PET = Pittsburgh Compound B Positron Emission Tomography, HADS = Hospital Anxiety and Depression Scale, NPI = Neuropsychiatric Inventory, WCST = Wisconsin Card Sorting Test, CFI = Cognitive Function Instrument, ADNI = Alzheimer’s Disease Neuroimaging Initiative. ↑ = high or elevated levels, ↓ = low levels.

**Table 3:** Summary of self-awareness, cognitive, and neurobiological changes across the Alzheimer’s disease continuum.

AD Stage	Self-Awareness Status	Principal Cognitive Findings	Principal Neurobiological Findings	Key Evidence
CU	Preserved awareness and/or hyperawareness; subtle awareness alterations may already emerge in biomarker-positive individuals	Intact cognition, subtle metamemory alterations preceding measurable cognitive decline	Early Aβ accumulation; subtle regional tau pathology; hippocampal hypometabolism; early DMN alterations involving medial prefrontal and posterior cingulate regions, with largely preserved structural integrity	Awareness alterations may be detectable before objective cognitive impairment. Longitudinal evidence (≈3–5 years) indicates that biomarker-positive individuals may subsequently develop declining awareness alongside increasing AD pathology.
SCD	Heterogeneous awareness profile; hyperawareness or emerging reduced awareness	Preserved objective cognition; heightened memory concern; subtle episodic memory and metamemory alterations	Increasing Aβ burden; emerging tau pathology; altered medial prefrontal, PCC and precuneus activity; early DMN dysfunction and altered self-referential processing	Evidence supports a transition from hyperawareness toward declining awareness as AD pathology accumulates. Greater cortical amyloid burden has been linked to increased memory concern, while reduced awareness, rather than subjective cognitive complaints alone, may better reflect underlying AD pathology despite preserved cognition.
MCI	Progressive decline in awareness, ranging from preserved insight to emerging anosognosia	Episodic memory impairment; executive dysfunction; impaired metacognitive updating; increasing participant–informant discrepancy	↑ Aβ; ↑ p-tau/t-tau; ↓ CSF Aβ42; hippocampal and medial temporal atrophy; FDG hypometabolism; PCC/ACC dysfunction; progressive disruption of DMN, SRN and memory-network connectivity	Findings, particularly from aMCI cohorts, point to declining awareness as a potential predictor of subsequent progression to AD, with reported effect estimates ranging from approximately 1.5- to 3-fold across cohorts. Higher conversion rates were observed among individuals with anosognosia, which has been reported to emerge approximately 3 years before dementia diagnosis.
AD	Predominant anosognosia with persistent overestimation of cognitive abilities	Global cognitive impairment; severe episodic memory deficits; executive dysfunction; impaired self-monitoring and failure to update self-evaluations	Extensive amyloid and tau pathology; widespread cortical atrophy; PCC/ACC hypometabolism; large-scale disruption of DMN, FPN, SN and self-referential networks	Persistent participant–informant discrepancies accompany advanced neurodegeneration. Many patients with AD seem to overestimate their memory performance and fail to update their self-evaluations, which has been linked to the progressive disruption of large-scale brain networks involved in self-referential processes.

CU = Cognitively Unimpaired; SCD = Subjective Cognitive Decline; MCI = Mild Cognitive Impairment, AD = Alzheimer’s Disease; Aβ = Amyloid-β; ACC = Anterior Cingulate Cortex; FDG = fluorodeoxyglucose; DMN = Default Mode Network; FPN = Frontoparietal Network; PCC = Posterior Cingulate Cortex; SN = Salience Network; SRN = Self-Referential Network. ↑ = high or elevated levels, ↓ = low levels.

## Data Availability

The raw data supporting the conclusions of this article will be made available by the authors on request.

## Supplementary Materials

The following supporting information can be downloaded at https://www.mdpi.com/article/10.3390/diagnostics16172791/s1, PRISMA 2020 Checklist; Supplementary Table S1. Excluded studies during title/abstract screening.; Supplementary Table S2. Excluded studies after full-text screening.

## Abbreviations

The following abbreviations are used in this manuscript:

ACC	Anterior Cingulate Cortex
AD	Alzheimer’s Dementia
aMCI	Amnestic MCI
CN	Cognitively Normal
CSF	Cerebrospinal Fluid
DMN	Default Mode Network
ERN	Early error detection
FDG	Fluorodeoxyglucose
FPN	Frontoparietal Network
MCI	Mild Cognitive Impairment
MMSE	Mini Mental State Examination
naMCI	Non-amnestic MCI
Pe	Error-related positivity
PET	Positron Emission Tomography
SCD	Subjective Cognitive Decline
SN	Salience Network
SRN	Self-Referential Network
