# Supplementary material for: Trajectories of Self-Awareness Across the Alzheimer’s Disease Spectrum: A Systematic Review of Its Potential Contribution to Early Diagnosis
Source: Diagnostics (Basel). 2026 Aug 30;16(17):2791. doi: 10.3390/diagnostics16172791 (PMC13564645; doi:10.3390/diagnostics16172791)
Supplement: Supplementary file 1 [file diagnostics-16-02791-s001.zip › Supplementary Table S1_ Excluded studies during title_abstract screening.pdf]

Supplementary Table S1. Exclude studies during title/abstract screening

First Author, Year	Doi	Reason for exclusion
Bigotto et al., 2026	10.1016/j.nicl.2024.103719	No relevant outcome
Sheng et al.,2024	10.1016/j.compbiomed.2024.108035	No relevant outcome
Liang et al.,2020	10.1016/j.brainresbull.2020.02.017	No relevant outcome
Zendehrouh et al, 2020	10.1016/j.nicl.2024.103719	No relevant outcome
Huang et al., 2024	10.1016/j.jfma.2023.11.012	No relevant outcome
Radakovic et al., 2017	10.3233/JAD-170292	No relevant outcome
Mitchell et al., 2024	10.1016/j.jnma.2024.01.017	No relevant outcome
Dovixio et al., 2025	10.1016/j.aip.2025.102367	No relevant outcome
Zhang et al., 2025	10.1016/j.brainres.2017.02.012	No relevant population
Eslami-Amirabadi et al., 2026	10.3389/fneur.2026.1724172	No relevant outcome
Luquiens et al., 2025	10.1016/j.jpsychires.2024.12.012	No relevant population
Wei et al., 2025	10.2196/72082	No relevant outcome
Ulep et al., 2018	10.1016/j.nurpra.2017.10.014	Theoretical paper
Orfei et al., 2018	10.1016/j.parkreldis.2018.04.015	No relevant population
Croff et al., 2024	10.1093/geront/gnae019	No relevant outcome
Richmond et al., 2022	10.1016/j.neuropsychologia.2022.108298	No relevant outcome
Sarabi et al., 2024	10.1016/j.neuroimage.2023.120504	No relevant outcome
Zhang et al., 2023	10.1016/j.neuroimage.2023.119951	No relevant outcome
Ferreira et al., 2020	10.6061/clinics/2020/e1863	No relevant outcome
Zhang et al.,, 2025	10.1016/j.apro.2025.100190	No relevant outcome
Fernandez- Mattarrubia, 2016	10.1016/j.jagp.2015.09.004	No relevant outcome
Byeon et al., 2021	10.30773/pi.2020.0364	Validation study
Umeda et al., 2024	10.1017/S1041610224000401	Validation study
Shao et al., 2025	10.1016/j.jamda.2025.105882	Validation study
Terry et al., 2024	10.1093/arclin/acae020	Validation study
Gerstenecker et al., 2019	https://doi.org/10.1002/gps.5118	No relevant outcome
Gonzalo-de Miguel et al., 2024	10.1016/j.schres.2024.07.040	No relevant population
Mortby et al., 2022	10.1016/j.jagp.2021.06.016	No relevant outcome
Oey et al., 2025	10.1016/j.jamda.2025.105797	No relevant population
Ready et al., 2016	10.1016/j.psychres.2016.07.046	No relevant outcome
Zhang et al., 2021	10.1016/j.gerinurse.2021.10.013	Review

Cook et al., 2025	10.2196/57018	No relevant outcome
Zwijsen et al., 2016	10.1017/S1041610216000351	Review
Bouldin et al., 2021	10.1017/S1041610220001635	No relevant outcome
Xu et al., 2025	10.1016/j.neuroscience.2025.04.031	No relevant outcome
Cloutier et al., 2021	10.1002/gps.5426	No relevant outcome
Vannini et al., 2025	10.1177/13872877251350138	Article commentary
Kandasamy et al., 2019	10.1016/j.jth.2019.03.005	No relevant outcome
Goldberg et al., 2021	10.1017/S1041610220000319	No relevant outcome
McDermott et al., 2019	10.1016/j.ynstr.2019.100162	No relevant outcome
Su et al., 2026	10.1177/13594575261424462	Review
Pusswald et al., 2016	10.1017/S1041610216001289	No relevant outcome
Chen et al, 2025	10.1016/j.brainresbull.2025.111320	No relevant outcome
Klainin-Yobas et al., 2019	10.1016/j.jocn.2019.05.031	No relevant outcome
Guan et al., 2024	10.1017/S1041610224000590	No relevant outcome
Banning et al., 2020	10.1016/j.jagp.2020.01.012	No relevant outcome
Vasta et al., 2018	10.1016/j.yebeh.2018.09.008	No relevant population
Ramabharathi et al., 2026	10.1016/j.pscychresns.2026.112269	No relevant outcome
Chien et al., 2024	10.2196/51987	No relevant outcome
Roehr et al., 2017	10.1017/S1041610217001399	No relevant outcome
vom Hofe et al., 2025	10.1016/j.jad.2025.119998	No relevant outcome
Vespa et al., 2022	10.1007/s40520-021-01907-x	No relevant outcome
Huang et al., 2019	10.1016/j.nicl.2018.101642	No relevant outcome
Fischer et al., 2026	10.1097/YCT.0000000000001229	Case study
Tian et al., 2023	10.1016/j.lanwpc.2023.100862	No relevant outcome
Desmarais et al., 2018	10.1017/S1041610217001260	No relevant outcome
Davis & Owens et al., 2021	10.3928/00989134-20201209-01	No relevant outcome
LeBlond et al., 2016	10.1016/j.cortex.2015.10.017	No relevant outcome
Phung et al. 2018	10.1016/j.jagp.2017.06.005	No relevant outcome
Fragkiadaki et al., 2018	10.1097/WAD.0000000000000254	No relevant outcome
Zhao et al., 2025	10.1093/braincomms/fcaf235	No relevant outcome
Greydanus et al., 2025	10.1016/j.disamonth.2025.101983	No relevant population
Dimitriadis et al., 2024	10.1016/j.compbiomed.2024.108862	No relevant population
Clevenger et al., 2025	10.1016/j.gerinurse.2024.11.020	No relevant outcome

Fnag et al., 2019	10.1016/j.archger.2019.02.001	No relevant outcome
Chen et al., 2023	10.1016/j.jth.2023.101710	No relevant outcome
Spens et al., 2026	10.2196/87295	No relevant outcome
Manera et al., 2020	10.1016/j.jagp.2019.07.014	No relevant outcome
Prasad et al., 2023	10.1016/j.disamonth.2022.101441	Review
Siddarth et al., 2020	10.1093/ajcn/nqz241	No relevant outcome
Roy et al., 2024	10.1016/j.bpsc.2024.04.002	No relevant outcome
Bassi et al., 2018	10.1016/j.gerinurse.2018.01.002	No relevant outcome
Creese et al., 2020	10.1017/S1041610219001200	No relevant population
Bond et al., 2016	10.3389/fnhum.2016.00097	No relevant outcome
Sheikh et al., 2018	10.1017/S104161021700151X	No relevant population
Amado et al., 2020	10.1016/j.bja.2020.04.072	No relevant population
Zhang et al., 2024	10.1016/j.bpsc.2024.04.004	No relevant outcome
Yue et al., 2025	10.1016/j.archger.2024.105623	No relevant outcome
Horne et al., 2022	10.1016/j.neuropsychologia.2022.108244	No relevant population
Steward et al., 2019	10.1016/j.neuropsychologia.2019.04.023	No relevant outcome
De Farias et al., 2021	10.1007/s12035-021-02411-z	No relevant outcome
Zheng et al., 2026	10.1016/j.bbih.2026.101176	Review and meta-analysis
Mitelpunkt et al., 2020	10.1038/s41598-020-57785-2	No relevant outcome
Cammisuli et al., 2016	10.1016/j.eurger.2016.01.002	Review
Ilardi et al., 2022	10.1007/s40520-021-01930-y	Review
Amanzio et al., 2018	10.1017/S1041610218000455	No relevant outcome
Holland et al., 2018	10.1093/cdn/nzy043	No relevant outcome
Estarellas et al., 2024	10.1002/gps.6112	No relevant outcome
Bahnfleth et al., 2021	10.1093/cdn/nzy043	No relevant population
Amanzio et al., 2016	10.1016/j.exger.2016.08.008	No relevant population
Wu et al., 2019	10.1016/j.bbr.2018.10.039	No relevant outcome
Saari et al., 2020	10.1016/j.jad.2020.07.100	No relevant outcome
Lloyd et al., 2026	10.1016/j.neuropsychologia.2026.109492	No relevant outcome
Paire-Ficout et al., 2018	10.3233/JAD-171031	No relevant outcome
Zhou et al., 2026	10.1016/j.neuroscience.2026.02.024	No relevant population
Nuber-Champier et al., 2022	10.1016/j.bbih.2022.100511	No relevant population
Diana et al., 2023	10.1016/j.jns.2022.120511	No relevant population

Shaked et al., 2019	10.1371/journal.pone.0222769	No relevant outcome
Harvey et al., 2023	10.1016/j.jagp.2023.07.009	No relevant population
Julayanont et al., 2020	10.1016/j.jns.2019.116523	No relevant population
Leow et al., 2026	10.1016/j.bpsc.2025.06.010	No relevant population
Alderson et al., 2018	10.1016/j.neuroimage.2018.08.033	No relevant outcome
Yu et al., 2022	10.1037/neu0000773	No relevant outcome
Kero et al., 2023	10.3233/JAD-221140	No relevant population
Lizuka et al., 2017	10.1016/j.pscychresns.2017.09.007	No relevant population
Marks et al., 2026	10.1016/j.arrct.2026.100656	No relevant outcome
Kumai et al., 2021	10.1159/000516656	No relevant outcome
Mendes et al., 2021	10.1016/j.jpsychires.2021.03.016	No relevant population
Small et al., 2018	10.1016/j.jagp.2017.10.010	No relevant outcome
Berry et al., 2017	10.1016/j.socscimed.2017.07.003	No relevant outcome
Waschkies et al., 2023	10.1002/gps.6007	No relevant outcome
Bracca et al., 2025	10.1016/j.jagp.2024.12.005	No relevant population
Kawasaki et al., 2016	10.1016/j.parkreldis.2015.11.015	No relevant population
Gabb et al., 2025	10.2196/72824	No relevant outcome
Kreshpa et al., 2024	10.3233/JAD-240430	No relevant outcome
Yasuno et al., 2025	10.1016/j.bbih.2025.101018	No relevant outcome
Villeneuve et al., 2019	10.1016/j.trci.2019.08.009	No relevant outcome
Pillai et al., 2018	10.1002/mdc3.12606	No relevant population
Zeng et al., 2025	10.1016/j.media.2024.103368	No relevant outcome
Bruton et al., 2021	10.1016/j.intell.2021.101540	No relevant outcome
Robert et al., 2018	10.1016/j.eurpsy.2018.07.008	No relevant outcome
Lazarou et al., 2019	10.1016/j.clinph.2019.07.004	Review
Li-Chay-Chung et al., 2024	10.1016/j.neuropsychologia.2024.108943	No relevant outcome
Torrente et al., 2025	10.1016/j.nrl.2023.03.009	No relevant outcome
Isaacs et al., 2024	10.1016/j.parkreldis.2024.106969	No relevant population
Etholen et al., 2024	10.1016/j.ypmed.2023.107830	No relevant outcome
Maier et al., 2023	10.1017/S0033291721002725	No relevant population
Li et al., 2026	10.1016/j.gerinurse.2026.103946	Review
Pujol et al., 2017	10.1016/j.sleep.2017.07.021	No relevant outcome
Bertrand et al., 2017	10.1016/j.cortex.2016.02.016	Theoretical

Klingbeil et al., 2020	10.1016/j.neuroimage.2019.116485	No relevant population
Beason-Held et al., 2021	10.1007/s11682-020-00279-6	No relevant outcome
Phillipi et al., 2017	10.1016/j.cortex.2017.06.019	Case study
Burley et al., 2025	10.2196/75816	No relevant population
Conlon et al., 2025	10.1016/j.socscimed.2025.118510	No relevant population
Alexander et al., 2025	10.1080/09602011.2024.2337152	Validation study
Raggi et al., 2017	10.1016/j.eurger.2017.01.008	No relevant outcome
Mondragon et al., 2019	10.1007/s11065-019-09410-x	Review
Huff et al., 2025	10.1016/j.ynirp.2025.100266	No relevant outcome
Hata et al., 2025	10.1016/j.clinph.2015.10.030	No relevant outcome
Uquillas, et al., 2020	10.1093/cercor/bhz217	No relevant outcome
Sunderaraman et al., 2022	10.1002/hbm.25747	No relevant outcome
Wang et al., 2025	10.1016/j.brainresbull.2025.111315	No relevant population
Koskas et al., 2018	10.1016/j.neurol.2017.05.012	No relevant outcome
Huang et al., 2023	10.1016/j.gerinurse.2023.01.018	No relevant outcome
Yokoi et al., 2026	10.1016/j.ebr.2026.100853	No relevant population
Rogers et al., 2026	10.2196/85626	No relevant outcome
Sejunaite et al., 2017	10.3233/JAD-170493	No relevant outcome
Manniet al., 2019	10.1016/j.sleep.2019.04.018	No relevant outcome
Arslan-Sarımehmetoğlu et al., 2024	10.1016/j.jvoice.2024.11.033	No relevant outcome
Vallejo et al., 2017	10.1016/j.chb.2017.01.021	Validation study
Kang et al., 2025	10.2196/69689	Validation study
Ilardi et al., 2026	10.1177/08919887251362470	No relevant outcome
Lou et al., 2024	10.1080/13607863.2024.2367038	No relevant outcome
Saez et al., 2021	10.1016/j.med.2021.12.002	No relevant outcome
Cai et al., 2022	10.1016/j.gerinurse.2022.08.001	No relevant population
Kim et al., 2022	10.1080/13607863.2021.1922602	No relevant outcome
Wei et al., 2025	10.1016/j.ctim.2025.103252	No relevant population
Batubara et al., 2023	10.1016/j.apnu.2023.04.011	Review
Conde-sala et al., 2016	10.1002/gps.4298	No relevant outcome
Rodrigues et al., 2020	10.1016/j.exger.2020.110890	No relevant outcome
Yan et al., 2024	10.1016/j.ijnurstu.2024.104902	No relevant outcome
Jacus et al., 2018	10.1016/j.neurol.2017.08.003	Validation study

Badal et al., 2025	10.1016/j.inpsyc.2024.100007	No relevant outcome
Watanabe et al., 2026	10.1177/25424823261437682	No relevant outcome
Yang et al., 2022	10.1016/j.jad.2022.03.014	No relevant population
Giacomucci et al., 2024	10.1016/j.cortex.2023.12.009	No relevant outcome
El Haj et al., 2017	10.1016/j.concog.2017.01.009	No relevant outcome
Gagnon et al., 2019	10.5664/jcsm.7664	No relevant population
Steward et al., 2016	10.1016/j.ijpsycho.2016.04.005	No relevant outcome
Fernández-Matarrubia et al., 2018	10.1002/gps.4695	No relevant population
Steward et al., 2019	10.1080/13803395.2019.1586839	No relevant outcome
Berry et al., 2025	10.1016/j.amjmed.2025.05.034	Case study
Aihara et al., 2025	10.1016/j.gerinurse.2025.03.032	Validation study
Yi et al., 2024	10.1016/j.ajp.2024.103924	Validation study
Torrente et al., 2025	10.1016/j.nrleng.2025.07.002	Validation study
Lim et al., 2026	10.2196/79386	No relevant outcome
Li et al., 2024	10.2196/48777	No relevant outcome
Curisic et al., 2022	10.2196/35442	No relevant outcome
Johar et al., 2023	10.1016/j.ypmed.2022.107390	No relevant population
Riaz et al., 2020	10.1016/j.jneumeth.2019.108506	No relevant population
Wang et al., 2026	10.1016/j.media.2025.103827	No relevant outcome
Scherling et al., 2016	10.3389/fnagi.2016.00120	No relevant outcome
Belli et al., 2020	10.3389/fpsyg.2020.553886	No relevant outcome
Lv et al., 2023	10.1016/j.archger.2023.105113	Meta-analysis
Martinez-Moreno et al., 2016	10.1017/S104161021600123X	No relevant outcome
Krejcí et al., 2026	10.1177/13872877251401620	No relevant outcome
Gold et al., 2020	10.1037/neu0000630	No relevant outcome
Köbe et al., 2016	10.1016/j.neuroimage.2015.09.050	No relevant outcome
Li et al., 2024	10.1016/j.yebeh.2024.110095	No relevant population
García-Alberca et al., 2017	10.1016/j.archger.2017.02.008	No relevant outcome
Delrue et al., 2021	10.1016/j.encep.2020.05.016	No relevant population
Zhornitsky et al., 2021	10.1016/j.pscychresns.2021.111380	No relevant outcome
Miebach et al., 2019	10.1093/geront/gnx208	No relevant population
Dubois et al., 2018	10.1016/S1474-4422(18)30029-2	No relevant outcome
Ilardi et al., 2026	10.1111/jnp.70008	No relevant outcome

Stojanovic et al., 2024	10.1007/s40520-023-02643-0	No relevant outcome
Mondragón et al., 2019	10.1159/000500183	No relevant outcome
Fischer et al., 2026	10.1016/j.jagp.2026.02.015	No relevant outcome
Terraciano et al., 2023	10.1016/j.jamda.2023.05.011	No relevant outcome
Tonga et al., 2016	10.1177/1533317515603958	Case study
Salmon et al., 2024	10.1016/j.cortex.2024.07.004	No relevant outcome
Date et al., 2024	10.1186/s13195-024-01605-6	No relevant outcome
Sheng et al. ,2025	10.1016/j.brainres.2025.149691	No relevant outcome
Zhu et al., 2025	10.1016/j.nicl.2025.103913	No relevant outcome
Zhezi et al., 2025	10.1016/j.sleep.2025.106741	No relevant population
Robles Bayon et al., 2017	10.1016/j.nrleng.2015.01.003	No relevant outcome
Bertrand et al., 2017	10.3389/fnagi.2017.00182	No relevant outcome
Mihaljević et al., 2021	10.1016/j.neucom.2021.01.138	No relevant outcome
Pellichero et al., 2021	10.1016/j.rehab.2020.03.003	No relevant outcome
Tahmasebi et al., 2020	10.1007/s12078-019-09267-7	No relevant outcome
Stites et al. 2017	10.1093/geronb/gbx100	No relevant outcome
Stites et al., 2023	10.1002/gps.5939	No relevant outcome
Azar et al., 2017	10.1016/j.jns.2017.04.009	No relevant population
El Haj et al., 2019	10.1016/j.archger.2019.01.011	No relevant outcome
Manning Franke et al., 2025	10.1016/j.clinph.2021.09.007	No relevant population
Coebergh et al., 2025	10.1016/j.ibneur.2025.01.006	No relevant outcome
Vora et al., 2025	10.1016/j.disamonth.2025.101863	Theoretical
Adedeji et al., 2025	10.1016/j.inpsyc.2024.100009	No relevant outcome
Ourry et al., 2021	10.1016/j.neuroimage.2021.117819	No relevant outcome
Shikimoto et al., 2021	10.1016/j.jpsychires.2021.07.026	No relevant outcome
Fawzy et al., 2025	10.1038/s41598-025-29569-z	No relevant outcome
Scarfo et al., 2024	10.2174/0115672050309014240705113444	No relevant outcome
Graham et al., 2020	10.1016/j.psychres.2019.112732	Review
Malhotra et al.,2025	10.1016/j.aip.2025.102299	No relevant outcome
