# Supplementary material for: Trajectories of Self-Awareness Across the Alzheimer’s Disease Spectrum: A Systematic Review of Its Potential Contribution to Early Diagnosis
Source: Diagnostics (Basel). 2026 Aug 30;16(17):2791. doi: 10.3390/diagnostics16172791 (PMC13564645; doi:10.3390/diagnostics16172791)
Supplement: Supplementary file 1 [file diagnostics-16-02791-s001.zip › Supplementary Table S2 _Excluded Studies after full text screening.pdf]

Supplementary Table S2. Excuded Studies after full text screening

| First Author, Year | DOI | Reason for exclusion | Justification |
|---|---|---|---|
| Amariglio et al., 2018 | 10.1016/j.trci.2018.08.005 | No relevant outcome | Self-awareness was not assessed using validated awareness measure; the study investigated subjective cognitive complaints in relation to amyloid burden. |
| Andrade et al., 2023 | 10.3389/fneur.2023.1239057 | Ineligible publication type | This was a theoretical article and did not include original empirical data. |
| Anjos et al., 2024 | 10.1002/gps.70007 | No relevant outcome | Self-awareness was not directly assessed as an outcome; the study investigated changes in subjective memory complaints. |
| Antoine et al., 2019 | 10.1002/hbm.24775 | No relevant outcome | Amyloid-positive MCI and AD participants were analysed as a single group, precluding stage-specific interpretation of awareness across the AD continuum. |
| Arola et al., 2023 | 10.1016/j.cccb.2023.100182 | No relevant population | The study focused on cerebral small vessel disease. |
| Bajic et al., 2021 | 10.1177/26331055211033869 | Ineligible publication type | This was a theoretical article and did not present original empirical data. |
| Ball et al., 2023 | 10.1136/bmjopen-2023-073205 | No relevant outcome | Self-awareness was not directly assessed as an outcome; the study investigated subjective cognitive decline. |
| Bertrand et al., 2016 | 10.1016/j.cortex.2016.09.001 | No relevant outcome | The study investigated the effect of experimentally induced mood on awareness of symptoms. |
| Bertrand et al., 2018 | 10.1037/neu0000458 | No relevant outcome | Self-awareness was not directly assessed as an outcome; the study investigated the neural correlates of experimental metamemory. |
| Bertrand et al., 2021 | 10.3233/JAD-201246 | No relevant outcome | The primary objective was to investigate the structural neural correlates of multidomain awareness in AD. |
| Brailean et al., 2019 | 10.1016/j.jpsychires.2018.12.005 | No relevant outcome | Self-awareness was not directly assessed as an outcome; the study investigated subjective memory complaints. |
| Calil et al., 2021 | 10.1002/gps.5521 | No relevant population | The study compared anosognosia between dementia with Lewy bodies and AD dementia. |
| Cappa et al., 2024 | 10.1002/alz.13905 | Ineligible publication type | This was a narrative review and did not include original empirical data. |
| Castrillo- Sanz et al., 2016 | 10.1016/j.nrleng.2015.03.011 | No relevant outcome | The study investigated the prevalence and clinical predictors of anosognosia in established AD disease. |
| Ceolin et al., 2025 | 10.1016/j.gerinurse.2025.103440 | No relevant outcome | The study focused on caregiver burden and coping strategies. |
| Chapman et al., 2018 | 10.1016/j.cortex.2018.01.019 | No relevant outcome | Self-awareness was not directly assessed as an outcome; the study investigated experimental mechanisms of memory and motor self-monitoring. |
| Chapman et al., 2022 | 10.3389/fnagi.2022.787552 | No relevant population | Population not confirmed to lie within the Alzheimer's disease continuum. |

| | | | |
|---|---|---|---|
| Chen et al., 2019 | 10.1016/j.dadm.2018.12.007 | No relevant outcome | Self-awareness was not directly assessed as an outcome; the study focused on subjective cognitive decline and metamemory beliefs. |
| Chio et al., 2018 | 10.1016/j.jfma.2017.02.015 | No relevant outcome | Self-awareness was not directly assessed as an outcome; the study evaluated the psychometric properties of the AD8 questionnaire. |
| Chao et al., 2021 | 10.14283/jpad.2020.61 | No relevant outcome | The primary objective was to investigate executive function as a predictor of subjective–objective memory discrepancy. |
| Coddaire et al., 2025 | 10.3389/fneur.2025.1666039 | No relevant outcome | Self-awareness was not directly assessed as an outcome; the study focused on awareness of functional abilities. |
| Contreras et al., 2017 | 10.1016/j.dadm.2016.12.004 | No relevant outcome | Self-awareness was not directly assessed as an outcome; the study investigated the association between subjective cognitive complaints and resting-state functional connectivity. |
| Cova et al., 2017 | 10.1177/1533317517725812 | No relevant outcome | The study used a single self-reported memory complaint item rather than a validated measure of awareness. |
| Denny et al., 2021 | 10.1016/j.gerinurse.2020.08.009 | No relevant outcome | Self-awareness was not directly assessed as an outcome; the study evaluated the psychometric utility of the AD8 discrepancy score in patients with established AD or mixed dementia. |
| Duran et al., 2022 | 10.1007/s11682-021-00627-0 | No relevant outcome | Self-awareness was not directly assessed as an outcome; the study assessed performance prediction/postdiction. |
| Flores-Vázquez et al., 2021 | 10.3389/fnagi.2021.739422 | No relevant outcome | Awareness was assessed exclusively using an informant-based measure not a discrepancy-based awareness measure. |
| Fogarty et al., 2017 | 10.1080/13607863.2016.1207750 | No relevant outcome | The study focused on awareness of executive dysfunction. |
| Gainotti et al., 2018 | 10.1016/j.bandc.2018.08.002 | Ineligible publication type | This was a review article and did not present original empirical data. |
| Giannouli et al., 2022 | 10.3390/neurolint14030051 | No relevant outcome | The study focused on awareness of financial capacity. |
| Hamilton et al, 2026 | 10.1017/S0033291725102997 | No relevant population | The primary analyses were conducted in a mixed MCI cohort (MCI due to AD and MCI with Lewy bodies). |
| Harris et al., 2026 | 10.1177/13872877251406623 | No relevant outcome | Self-awareness was not directly assessed as an outcome; the study focused on experimental metamemory and confidence judgments. |
| Harvey et al., 2025 | 10.1016/j.inpsyc.2024.100025 | No relevant outcome | Self-awareness was not directly assessed as an outcome; the study investigated self-assessment of cognitive performance. |
| Haussmann et al., 2018 | 10.1177/1533317518775033 | No relevant outcome | Self-awareness was not directly assessed as an outcome; the study investigated subjective memory impairment and hippocampal cortical thickness. |
| Jacus et al., 2017 | 10.1002/brb3.661 | No relevant outcome | Self-awareness was not directly assessed as an outcome; the study focused on the association of awareness with apathy and depression. |
| Jacus et al., 2022 | 10.3233/JAD-215550 | No relevant outcome | The study focused on awareness of apathy. |
| Jiang et al., 2023 | 10.1002/alz.13068 | No relevant outcome | Self-awareness was not directly assessed as an outcome. |
| Klein et al., 2023 | 10.1016/j.brs.2023.04.007 | Ineligible study design | This was a qualitative study and did not provide quantitative data. |

| | | | |
|---|---|---|---|
| Kühles et al., 2024 | 10.7345/prolog-2402084 | No relevant outcome | The study focused on the development and validation of a self-assessment questionnaire. |
| La Joie et al., 2016 | 10.1016/j.dadm.2016.12.005 | No relevant outcome | Self-awareness was not directly assessed as an outcome; the study investigated subjective cognitive complaints. |
| Lacerda et al., 2017 | 10.1097/WAD.0000000000000210 | No relevant outcome | The study investigated multidimensional awareness rather than awareness of cognitive decline. |
| Lacerda et al., 2021 | 10.1002/gps.5495 | No relevant outcome | The study assessed multiple domains of awareness rather than awareness of cognitive decline. |
| Li et al., 2022 | 10.1016/j.nicl.2022.103255 | No relevant population | Population included not confirmed to lie within the Alzheimer's disease continuum. |
| López-Martos et al., 2026 | 10.1016/j.tjpad.2026.100612 | No relevant outcome | Self-awareness was not directly assessed as an outcome; the study focused on objectively defined subtle cognitive decline using AD biomarkers. |
| Mendes et al., 2019 | 10.3233/JAD-190414 | No relevant outcome | Self-awareness was not directly assessed as an outcome; the study investigated subjective memory complaints in relation to amyloid status. |
| Oba et al., 2019 | 10.1080/13607863.2018.1442411 | No relevant outcome | The study investigated the influence of depressive symptoms on anosognosia. |
| Papirovitz et al., 2021 | 10.2174/1567205018666210416095437 | No relevant outcome | Self-awareness was not directly assessed as an outcome; the study investigated subjective cognitive complaints. |
| Perrotin et al., 2017 | 10.1016/j.jalz.2016.08.011 | No relevant outcome | The study focused on subjective cognitive decline rather than discrepancy-based awareness measures. |
| Prigatano et al., 2024 | 10.3389/fnagi.2023.1325231 | Ineligible publication type | This was a narrative review and did not include original empirical data. |
| Quaranta et al., 2023 | 10.1037/neu0000896 | No relevant outcome | The study assessed self-awareness of empathy. |
| Regueira et al., 2026 | 10.1080/13607863.2025.2552425 | No relevant outcome | Self-awareness was assessed using patient–informant discrepancy in subjective memory complaints. |
| Ruttala et al., 2022 | 10.46947/joaasr442022460 | No relevant outcome | Self-awareness was not directly assessed as an outcome; the study investigated diffusion tensor imaging alterations in AD. |
| Sánchez-Benavides et al., 2018 | 10.3233/JAD-180378 | No relevant population | The study population was not restricted to the AD continuum. |
| Savla et al., 2016 | 10.1037/fsh0000230 | No relevant outcome | The study focused on patient–caregiver concordance in the perception of cognitive and functional deficits and dyadic coping. |
| Serino et al., 2017 | 10.3233/JAD-160676 | Ineligible publication type | This was a theoretical article and did not include original empirical data. |
| Silva et al., 2017 | 10.1080/13803395.2016.1231799 | No relevant outcome | The study focused on the effects of memory training on memory self-awareness. |

| | | | |
|---|---|---|---|
| Tagai et al., 2018 | 10.1111/psyg.12287 | No relevant outcome | Participants with MCI due to AD and mild AD analysed as a single group, precluding stage-specific interpretation of awareness. |
| Tondelli et al., 2021 | 10.3389/fpsyt.2021.658934 | No relevant population | The study included mixed dementia etiologies and primarily investigated the association between anosognosia and NPS. |
| Tondelli et al., 2022 | 10.3389/fnagi.2022.816648 | No relevant outcome | Primary focus was implicit self-awareness rather than awareness of cognitive decline |
| Topiwala et al., 2021 | 10.1016/j.jagp.2020.07.002 | No relevant outcome | Self-awareness was not directly assessed as an outcome; the study investigated subjective cognitive complaints. |
| Turró-Garriga et al., 2016 | 10.3233/JAD-150706 | No relevant outcome | The study focused on the clinical course and predictors of anosognosia in established AD. |
| Valotassiou et al., 2022 | 10.3390/diagnostics12051136 | No relevant population | The study included mixed dementia syndromes. |
| Vannini et al., 2020 | 10.1001/jamanetworkopen.2020.27472 | No relevant population | The study included only individuals with autosomal dominant AD (PSEN1 E280A mutation). |
| Viviano et al., 2019 | 10.1016/j.neuroimage.2018.10.015 | No relevant outcome | Self-awareness was not directly assessed as an outcome; the study focused on functional connectivity and working memory. |
| Verhülsdonk et al., 2017 | 10.1097/wad.0000000000000169 | No relevant outcome | The primary objective was to investigate the contribution of repressive coping style to anosognosia |
| Wang et al., 2024 | 10.3389/fnagi.2024.1335878 | No relevant outcome | The study investigated the association between anosognosia and NPS. |
| Wilson et al., 2018 | 10.1037/neu0000437 | No relevant population | The study included mixed dementia etiologies, and the MCI group was not defined as MCI due to AD. |
| Yoon et al., 2017 | 10.1177/0891988717700508 | No relevant outcome | The study investigated the relationship between anosognosia, NPS and disease severity in early-onset AD rather than the assessment of awareness across the AD continuum. |
